# Trace elements in hemodialysis patients: a systematic review and meta-analysis

**DOI:** 10.1186/1741-7015-7-25

**Published:** 2009-05-19

**Authors:** Marcello Tonelli, Natasha Wiebe, Brenda Hemmelgarn, Scott Klarenbach, Catherine Field, Braden Manns, Ravi Thadhani, John Gill

**Affiliations:** 1 Department of Medicine, University of Alberta, Edmonton, Alberta, Canada; 2 Department of Medicine, University of Calgary, Alberta, Canada; 3 Department of Agricultural, Food and Nutritional Science, University of Alberta, Edmonton, Alberta, Canada; 4 Department of Medicine, Harvard Medical School, Boston, Massachusetts, USA; 5 Department of Medicine, University of British Columbia, Vancouver, British Columbia, Canada

## Abstract

**Background:**

Hemodialysis patients are at risk for deficiency of essential trace elements and excess of toxic trace elements, both of which can affect health. We conducted a systematic review to summarize existing literature on trace element status in hemodialysis patients.

**Methods:**

All studies which reported relevant data for chronic hemodialysis patients and a healthy control population were eligible, regardless of language or publication status. We included studies which measured at least one of the following elements in whole blood, serum, or plasma: antimony, arsenic, boron, cadmium, chromium, cobalt, copper, fluorine, iodine, lead, manganese, mercury, molybdenum, nickel, selenium, tellurium, thallium, vanadium, and zinc. We calculated differences between hemodialysis patients and controls using the differences in mean trace element level, divided by the pooled standard deviation.

**Results:**

We identified 128 eligible studies. Available data suggested that levels of cadmium, chromium, copper, lead, and vanadium were higher and that levels of selenium, zinc and manganese were lower in hemodialysis patients, compared with controls. Pooled standard mean differences exceeded 0.8 standard deviation units (a large difference) higher than controls for cadmium, chromium, vanadium, and lower than controls for selenium, zinc, and manganese. No studies reported data on antimony, iodine, tellurium, and thallium concentrations.

**Conclusion:**

Average blood levels of biologically important trace elements were substantially different in hemodialysis patients, compared with healthy controls. Since both deficiency and excess of trace elements are potentially harmful yet amenable to therapy, the hypothesis that trace element status influences the risk of adverse clinical outcomes is worthy of investigation.

## Background

Hemodialysis is the most common form of treatment for end-stage renal disease (ESRD), and is associated with considerable morbidity and mortality due to accelerated cardiovascular disease and infection. Despite the well-documented burden of disease, much remains to be learned about how best to prevent these complications of hemodialysis.

Hemodialysis removes uremic toxins primarily by allowing equilibration of plasma and dialysate across a semi-permeable membrane. Dialysate is created by adding carefully regulated quantities of biologically essential ions such as potassium, sodium, bicarbonate, and calcium to water that has been treated to reduce solutes to very low levels. The dialysate concentration of other substances such as trace elements is not routinely manipulated. Substances that have lower concentrations in dialysate than in blood tend to be removed by dialysis. Although this is appropriate in the case of uremic toxins, it may lead to depletion of biologically essential substances. Besides the potential for ongoing removal of trace elements by dialysis, hemodialysis patients are at risk for low dietary intake of such substances due to uremia-related anorexia and dietary restrictions.

Hemodialysis patients are exposed to very high volumes (>300 liters/week) of dialysate. Therefore, even minute levels of toxic substances in source water could lead to tiny concentration gradients between blood and dialysate, which in turn could lead to clinically relevant toxicity. Substances present in dialysate but not in blood will tend to accumulate in the patient, and the lack of renal clearance in hemodialysis patients might theoretically lead to toxicity of ingested trace elements even when they are not present in dialysate. Thus, hemodialysis patients are at theoretical risk for both deficiency and accumulation of trace elements, depending on dietary intake, removal by dialysis, the composition of the source water used for hemodialysis, and residual kidney function [1-3].

Deficiency of essential trace elements (such as zinc or selenium) and excess of potentially harmful trace elements (such as lead or arsenic) are both known to have adverse consequences in the general population [4-10]. Although not established, it is plausible that disordered trace element nutritional status (if present) would contribute to morbidity and mortality among hemodialysis patients as well. However, the incidence of abnormal trace element status in dialysis patients has not been comprehensively studied. We performed a systematic review to compare trace element status between hemodialysis patients and healthy controls.

## Methods

### Data sources and searches

This systematic review is reported according to published guidelines [11]. An expert librarian conducted a comprehensive search to identify all relevant studies regardless of language or publication status. Three electronic databases, MEDLINE (1966 to 13 April 2008), EMBASE (1988 to 13 April 2008), and the Cochrane Library (13 April 2008) were searched. The detailed search strategies are included in Additional file 1. A subject specialist and a methodologist screened each citation or abstract. Any study considered potentially relevant by at least one reviewer was retrieved for further review.

### Study selection

The full text of each potentially relevant study was independently assessed by two reviewers for inclusion in the review using predetermined eligibility criteria on a pre-printed form. Studies were eligible for inclusion if they measured trace element concentrations in both a chronic hemodialysis population and a healthy control population. We selected the following trace elements for study *a priori *based on their known or suspected potential to influence health, and after consideration of existing standards for hemodialysis water quality [12]: antimony, arsenic, boron, cadmium, chromium, cobalt, copper, fluorine, iodine, lead, manganese, mercury, molybdenum, nickel, selenium, tellurium, thallium, vanadium, and zinc. Only studies that measured trace element status in whole blood, serum, or plasma were included. Disagreements were resolved by discussion and consultation with a third party. Disagreements arose with 6% of the articles (*κ *= 0.88).

### Data extraction and quality assessment

We assessed and reported the study quality of included studies using the Downs and Black checklist [13]. Two reviewers independently assessed each included study, and resolved disagreements with the aid of a third party through consensus. An average of 18% of disagreements on quality items occurred. Study characteristics and data of interest were pre-specified, and were recorded in a purpose-built database. One reviewer extracted the data. A second reviewer checked the data for accuracy.

### Data synthesis and analysis

We analyzed data using Review Manager 4.2.10 (Oxford, UK) and Stata 10.0 (College Station, Texas, USA). We calculated standardized mean differences (SMD) [14]; the hemodialysis population mean minus the control population mean divided by their pooled standard deviation (SD). By presenting the differences in means relative to variability, we removed the heterogeneous effects of both unit and assay, with SMD of 0.2, 0.5, and 0.8 units of SD representing small, medium, and large sizes of effect, respectively [15]. One study [16] reported geometric means rather than arithmetic means, requiring us to standardize the differences in log means rather than the differences in mean [17].

We assessed heterogeneity using the I^2 ^statistic [18,19], and found considerable between-study heterogeneity (*I*^2 ^>85%). Therefore, the primary method of pooling data across studies used a qualitative approach (vote counting [20]; used to tally the number of studies finding that the level of a particular trace element was lower or higher in hemodialysis patients than in controls). We used the sign test to determine whether the vote count tally was statistically significant (provided that there were at least three studies which reported results for the element in question). To provide guidance on the relative magnitude of the differences between hemodialysis patients and controls, we also report random-effects estimates of the pooled SMD for elements where the sign test indicated that levels were significantly different between these two populations. Given the presence of large heterogeneity, we did not formally assess for the presence of publication bias [21].

Statistical sources of heterogeneity were explored using weighted least squares meta-regression [22]. The following study-level variables were considered: sample source (whole blood, serum, plasma), mean duration of hemodialysis treatment (in months), continent on which the study was performed (Americas/Europe versus other) and measurement technique (absorption spectroscopy versus other). The effect of sample size was also explored given the tendency for SDs to be underestimated [23], and therefore SMDs overestimated, when sample sizes are small (*N *<30).

## Results

### Search yield

**Figure 1:**
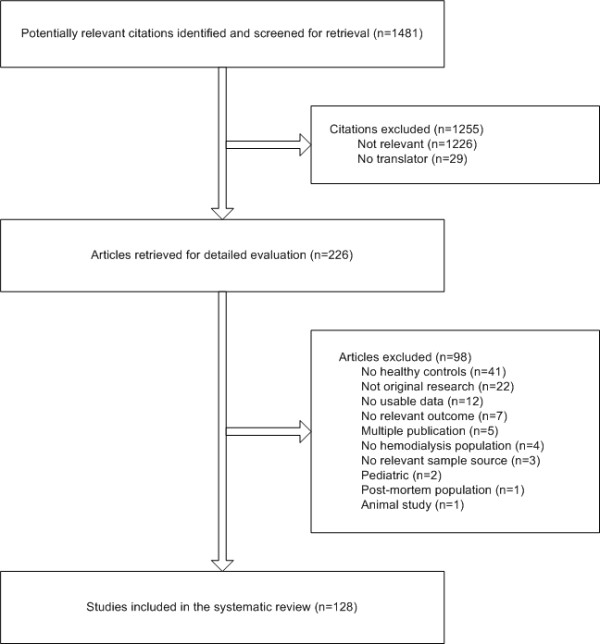
**Flow diagram of study selection**.

From 1,481 identified citations and abstracts, 226 full articles were retrieved for detailed evaluation (Figure 1). Of these, 128 studies [2,16,24-149] reported relevant data and were included in this review. Most studies were conducted in Europe (47%), Asia (30%), and North America (14%). Seventy-four percent of data came from cross-sectional studies; the remaining data from baseline assessments of randomized controlled trials and prospective cohort studies. Most studies used absorption spectroscopy, emission spectroscopy or neutron activation to measure trace element levels in biological specimens (Additional file 2). Multiple variants of absorption spectroscopy were used.

### Populations studied

Sample sizes of hemodialysis patients ranged from 6 to 456 (median 24; Additional file 2). Mean ages ranged from 32 to 74 and percent male ranged from 0% to 100%. Little information was reported on the primary cause of ESRD, co-morbidities, or membrane properties. Control group sample sizes ranged from 5 to 490 (median 28). Mean ages ranged from 28 to 74 and percent male ranged from 0% to 100%. Hospital or laboratory staff (13%), blood donors (7%), non-renal patients (6%), or people drawn from the general population in the same geographic region (6%) were the most commonly specified source populations for control groups. Results of quality assessment are reported in Additional file 2. Time spent on dialysis (39%), description of control participants (29%), and eligibility criteria (20%) were poorly reported. Adjustment or matching for age and sex was infrequent (20%). Technique was well reported (98%), but consideration for measurement error was poor (34%).

### Trace element status

**Figure 2:**
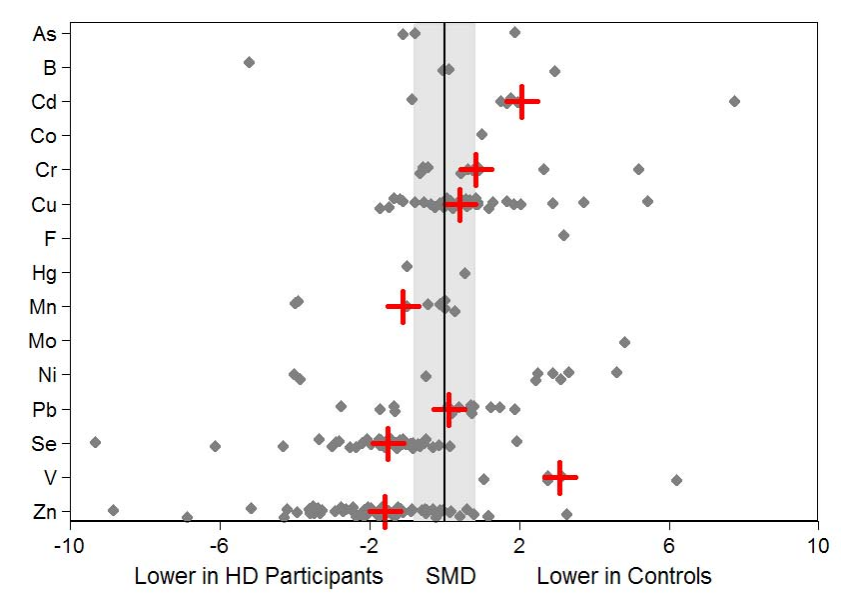
**Standardized mean differences of trace element concentrations: hemodialysis participants versus healthy controls**. The small solid gray diamonds represent the standardized mean difference (SMD) in trace element concentrations between hemodialysis patients and controls (for each individual study). The large red crosses represent the random-effects pooled SMDs. Results were pooled only for trace elements that were measured in at least three studies and for which the sign test was statistically significant. The shaded gray region denotes SMD representing differences between hemodialysis patients and controls, which are moderate or small (<0.8 standard deviation). Data points outside the gray region represent large differences between hemodialysis patients and controls (SMD ≥0.8 standard deviation).

Data were available for arsenic, boron, cadmium, chromium, cobalt, copper, fluorine, lead manganese, mercury, molybdenum, nickel, selenium, vanadium, and zinc. The pooled results comparing trace element status for hemodialysis patients with controls are presented in Table 1 and Figure 2. Results stratified by sample source (whole blood, serum, or plasma), or repeated using a fixed-effect model were consistent (data not shown). Available data suggested that levels of cadmium, chromium, copper, lead, and vanadium were higher and that levels of manganese, selenium, and zinc were lower in hemodialysis patients, compared with controls (Table 2). The magnitude of these differences was large (>0.8 SD units) for cadmium, chromium, vanadium, selenium, zinc, and manganese.

**Table 1:** Trace element concentrations independent of sample source

Element	Number of cohorts^b^	Number of hemodialysis participants and control participants	Pooled^a ^ SMD	I^2^, %	Range of SMDs	Number of studies significantly favouring lower concentrations in hemodialysis participants	Number of studies significantly favouring lower concentrations in control participants	Number of studies with non-significant findings
Arsenic	3	110/54	-	96	(-1.23, 1.77)	1	1	1
Boron	4	69/494	-	96	(-5.21, 2.96)	1	1	2
Cadmium	6	722/968	2.07	98	(-1.01, 7.7)	1	5^c^	0
Chromium	11	330/635	0.84	95	(-0.66, 5.14)	0	6^c^	5
Cobalt	1	7/9	-	-	-	0	0	1
Copper	42	1712/1444	0.52	93	(-1.74, 5.28)	6	16^c^	20
Fluorine	1	7/8	-	-	-	0	1	0
Lead	14	1217/1751	0.11	96	(-2.89, 1.73)	4	5^c^	5
Manganese	8	399/522	-0.7	96	(-3.96, 0.29)	4^c^	0	4
Mercury	2	607/264	-	99	(-0.94, 0.49)	1	1	0
Molybdenum	1	14/59	-	-	-	0	1	0
Nickel	9	369/323	-	99	(-3.96, 4.67)	3	6	0
Selenium	46	1496/1443	-1.50	91	(-9.16, 1.97)	37^c^	1	8
Vanadium	5	112/137	3.07	87	(1.18, 6.28)	0	5^c^	0
Zinc	74	2515/2699	-1.61	95	(-8.99, 3.24)	56^c^	5	13

^a^Pooled SMDs using the random-effects method; this estimate indicates the average effect of all included studies. Negative values indicate lower concentrations in the hemodialysis participants and positive values indicate lower concentrations in the control participants. Pooled SMDs are reported only when vote counting results are significant.

^b^Some studies counted more than once if results reported by strata, for example, women and men separately.

^c^Vote counting sign test results were significant at the *P *≤ 0.05 level. Applied to comparisons with a minimum of three studies.

SMD = standardized mean difference.

**Table 2:** Summary of findings

	Probably accumulates in hemodialysis patients	May accumulate in hemodialysis patients	Probably deficient in hemodialysis patients	Insufficient information
Cadmium	X			
Chromium	X			
Nickel	X			
Vanadium	X			
Copper		X		
Lead		X		
Manganese			X	
Selenium			X	
Zinc			X	
Antimony				X
Arsenic				X
Boron				X
Cobalt				X
Fluorine				X
Iodine				X
Mercury				X
Molybdenum				X
Tellurium				X
Thallium				X

Available data for arsenic, boron, cobalt, fluorine, mercury, molybdenum, and nickel were either too limited or too heterogeneous to report. No studies reported data on antimony, fluorine, iodine, tellurium, and thallium.

### Meta-regression

We attempted to identify potential explanations for the observed between-study heterogeneity using meta-regression. None of the characteristics considered (sample source, duration of hemodialysis treatment, continent on which the study was performed, or measurement technique) significantly modified the association between hemodialysis treatment and the blood levels of the trace elements studied (data not shown).

## Discussion

We found that hemodialysis patients appear to have lower levels of zinc and selenium than people in the general population. Zinc deficiency is a leading cause of disease in developing countries [4], and is associated with delayed wound healing [5], and immune deficiency characterized by impaired cell proliferation, abnormal T-cell function, defective phagocytosis, and abnormal cytokine expression [150,151], all of which might contribute to the excess risk of infection observed in hemodialysis patients [152-157]. Zinc deficiency may also cause or contribute to a number of relatively non-specific conditions commonly observed in hemodialysis patients, including anorexia [158], dysgeusia [159], and impaired cognitive function [160].

Although the biological significance of low blood selenium concentrations is less clear, severe selenium deficiency leads to sudden death and cardiomyopathy in the general population [6,7,161]. Lower levels of serum selenium without severe deficiency have been associated with hypertension [162], heart failure [163,164], and coronary disease [165] in the general population, and with cardiomyopathy among dialysis patients [1,166,167]. Finally, mild selenium deficiency appears to increase susceptibility to oxidant stress [168,169], which may be relevant to hemodialysis patients in whom oxidative stress is markedly increased [170-172].

In addition to the lower levels of selenium and zinc, hemodialysis patients seem to have higher average levels of certain trace elements compared with healthy controls. It was not possible to compare absolute levels of trace elements across studies (given the different techniques and specimens), and therefore we could not estimate the proportion of hemodialysis patients who have trace element levels in the toxic range. However, for many of the elements, the SMD for the differences between hemodialysis patients and controls exceeded 0.8 SD, which is considered to be a large between-group effect by authorities [15].

For most of the trace elements studied, the biological significance of higher blood levels is unclear. However, excessive levels of lead or arsenic in blood and other tissues are known to be potentially harmful. For example, minor elevations in whole blood lead levels are associated with impaired cognitive function [173,174], impaired hemoglobin synthesis, and hypertension [175,176]. Cerebrovascular disease and renal insufficiency [177-179] also appear to be more frequent at higher levels of blood lead, perhaps mediated by higher blood pressure. Thus, higher blood levels of lead may increase the risk of cardiovascular disease, although the mechanism remains unclear. Similarly, inorganic arsenic causes tissue damage by multiple mechanisms including oxidative injury [180,181], inhibition of DNA repair [182], and chromosomal damage (deletion, aneuploidy [183]), and higher levels of arsenic are associated with increased risk of peripheral vascular disease [184,185]. Animal studies suggest that low selenium status may predispose to arsenic toxicity [186], and underweight or malnourished humans may also be at increased risk [187], suggesting that hemodialysis patients may be at higher than average risk of arsenic toxicity.

### Implications of the findings

Although the concentration of some trace elements in hemodialysis source water is monitored annually in accordance with federal regulation [188], blood or body levels of these substances are rarely (if ever) measured in hemodialysis patients. The potential for the accumulation of trace elements to cause harm in hemodialysis patients is exemplified by aluminum. Aluminum toxicity led to serious toxicity (anemia, disabling encephalopathy, neuropathy and severe symptomatic bone disease) in dialysis patients prior to the recognition that aluminum in dialysate and oral medications was responsible [189-193]. It is difficult to overstate the importance of this discovery for nephrology practice, which led to the elimination of aluminum-related toxicity (and substantial improvements in patient outcomes) within a few years. Today, clinically obvious toxicity due to accumulation of aluminum is exceedingly rare in hemodialysis patients, and the existing regulation [188] has been remarkably effective at reducing the risk of acute toxicity from excess trace elements such as fluorine [194-196]. However, the possibility that other trace elements may accumulate in patients with kidney failure and cause unrecognized chronic toxicity has received surprisingly little attention.

Oral trace element supplements are readily available, and oral zinc and selenium preparations have been shown to increase blood levels of these elements in dialysis patients [197-200]. However, few data describe the impact of trace element supplementation on clinical outcomes in hemodialysis patients, despite the fact that correction of zinc deficiency is beneficial in the general population [5,201-204], including significantly reducing the risk of infection and all-cause death [205-211]. Data examining the relation between trace element status and clinical outcomes in hemodialysis patients are similarly scarce. One relatively small (*N *= 265) study published in the Lithuanian language shows a significant association between lower plasma zinc levels and the risk of infection [212]. Our data suggest that future studies should investigate the link between zinc or selenium status and clinical outcomes in dialysis patients, in whom the risk of infection is dramatically elevated compared with people with normal kidney function [152-157].

### Limitations

The available literature has several potentially important limitations. First, study quality was moderate to poor, and many of the studies were relatively small. Second, the available data include multiple different analytical techniques, study populations, control groups, and specimen types (whole blood, serum, plasma). Perhaps for this reason, there was substantial between-study heterogeneity in the results, meaning that the extent to which trace element levels are higher or lower in hemodialysis patients cannot be estimated with certainty. We were unable to identify statistical sources of this heterogeneity, perhaps because we only had access to study-level (rather than patient-level) data. Despite the heterogeneity, for several of the elements studied, the great majority of studies found differences between hemodialysis patients and controls. Since the same technique and specimen types were used for both patients and controls, it is unlikely that these differences are spurious. However, it remains possible that important differences exist for other substances known or suspected to influence health. Third, studies generally did not report data on dietary intake of trace elements and thus we cannot assess whether reduced consumption of foods containing zinc and selenium was responsible for the lower levels of these elements among hemodialysis patients. Fourth, although we found large differences between the blood levels of certain trace elements in hemodialysis patients and those in control participants, the clinical significance of this finding remains to be confirmed. Fifth, although we studied blood levels of trace elements, it is possible that bone or other compartments may better reflect trace element body status, especially for heavy metals such as lead [213]. Sixth, although simultaneous derangement of multiple trace elements (for example, low selenium and excess arsenic) may have synergistic toxicity [186], the nature of the available data precluded us from determining how frequently this occurs in hemodialysis patients. Finally, although all systematic reviews have potential limitations, we conducted and reported this analysis according to published guidelines aimed at reducing bias [11]. Nonetheless, as with all systematic reviews, the strength of our conclusions is influenced by the quality of the studies on which they are based.

## Conclusion

In summary, we found that average blood concentrations of biologically important trace elements were substantially different in hemodialysis patients, compared with healthy controls. Since both deficiency and excess of trace elements are potentially amenable to therapy, the hypothesis that trace element status influences the risk of adverse clinical outcomes appears worthy of investigation, especially when one considers the experience of the nephrology community with aluminum.

## Abbreviations

ESRD: end-stage renal disease; SD: standard deviation; SMD: standard mean differences

## Competing interests

The authors declare that they have no competing interests.

## Authors' contributions

MT contributed to the conception and design, study selection, interpretation of data, and drafted the manuscript. NW managed the project, contributed to the conception and design, managed the acquisition of data (study selection, quality assessment, data extraction), conducted the analysis, and contributed to the drafting of the manuscript. BH, SK, CF, BM, RT, and JG contributed to the conception and design, interpretation of data, and revised the manuscript critically for important intellectual content. All authors approved the final manuscript.

## Pre-publication history

The pre-publication history for this paper can be accessed here:



## Supplementary Material

Additional file 1 **Appendix**. Literature search strategies. Click here for file

Additional file 2 **Appendix Table S1 and S2**. Table S1 – Description of included studies. Table S2 – Quality assessment of included studies. Click here for file

## References

[B1] Zima T, Tesar V, Mestek O, Nemecek K (1999). Trace elements in end-stage renal disease. 2. Clinical implication of trace elements. Blood Purif.

[B2] Zima T, Mestek O, Nemecek K, Bartova V, Fialova J, Tesar V, Suchanek M (1998). Trace elements in hemodialysis and continuous ambulatory peritoneal dialysis patients. Blood Purif.

[B3] D'Haese PC, De Broe ME (1996). Adequacy of dialysis: trace elements in dialysis fluids. Nephrol Dial Transplant.

[B4] Shrimpton R, Gross R, Hill I, Young M (2005). Zinc deficiency: what are the most appropriate interventions?. BMJ.

[B5] Prasad AS (1988). Zinc in growth and development and spectrum of human zinc deficiency. J Am Coll Nutr.

[B6] Burk RF (1978). Selenium in nutrition. World Rev Nutr Diet.

[B7] Ge K, Xue A, Bai J, Wang S (1983). Keshan disease – an endemic cardiomyopathy in China. Virchows Arch A Pathol Anat Histopathol.

[B8] Salonen JT, Alfthan G, Huttunen JK, Pikkarainen J, Puska P (1982). Association between cardiovascular death and myocardial infarction and serum selenium in a matched-pair longitudinal study. Lancet.

[B9] Suadicani P, Hein HO, Gyntelberg F (1992). Serum selenium concentration and risk of ischaemic heart disease in a prospective cohort study of 3000 males. Atherosclerosis.

[B10] Moore JA, Noiva R, Wells IC (1984). Selenium concentrations in plasma of patients with arteriographically defined coronary atherosclerosis. Clin Chem.

[B11] Stroup DF, Berlin JA, Morton SC, Olkin I, Williamson GD, Rennie D, Moher D, Becker BJ, Sipe TA, Thacker SB (2000). Meta-analysis of observational studies in epidemiology: A proposal for reporting. Meta-analysis Of Observational Studies in Epidemiology (MOOSE) group. JAMA.

[B12] Association for the Advancement of Medical Instrumentation (2006).

[B13] Downs SH, Black N (1998). The feasibility of creating a checklist for the assessment of the methodological quality both of randomised and non-randomised studies of health care interventions. J Epidemiol Community Health.

[B14] Hedges LV, Olkin I (1985). Estimation of a single effect size: parametric and nonparametric methods. Statistical Methods for Meta-analysis.

[B15] Cohen J (1988). Statistical Power Analysis for the Behavioural Sciences.

[B16] Usuda K, Kono K, Iguchi K, Nishiura K, Miyata K, Shimahara M, Konda T, Hashiguchi N, Senda J (1996). Hemodialysis effect on serum boron level in the patients with long term hemodialysis. Sci Total Environ.

[B17] Deeks JJ, Higgins JPT, Altman DG, Higgins JPT, Green S (2008). Analysing data and undertaking meta-analyses. Cochrane Handbook for Systematic Reviews of Interventions Version 500 (updated February 2008).

[B18] Higgins JPT, Thompson SG (2002). Quantifying heterogeneity in a meta-analysis. Stat Med.

[B19] Higgins JPT, Thompson SG, Deeks JJ, Altman DG (2003). Measuring inconsistency in meta-analyses. BMJ.

[B20] Bushman BJ, Cooper H, Hedges LV (1994). Vote-counting procedures in meta-analysis. The Handbook of Research Synthesis.

[B21] Song F, Eastwood AJ, Gilbody S, Duley L, Sutton AJ (2000). Publication and related biases. Health Technol Assess.

[B22] Thompson SG, Higgins JP (2002). How should meta-regression analyses be undertaken and interpreted?. Stat Med.

[B23] Pearson ES (1960). The Application of Statistical Methods to Industrial Standardization and Quality Control.

[B24] Bober J, Kwiatkowska E, Kedzierska K, Olszewska M, Golebiewska E, Stachowska E, Kucharska E, Ciechanowski K, Chlubek D (2007). Influence of glucose in the dialysate on the activity of erythrocyte-glutathione-peroxidase and blood selenium concentration in hemodialyzed patients. Arch Med Res.

[B25] Zagrodzki P, Barton H, Walas S, Folta M, Stompor T, Janusz-Grzybowska E, Drozdz M, Sulowicz W (2007). Selenium status indices, laboratory data, and selected biochemical parameters in end-stage renal disease patients. Biol Trace Elem Res.

[B26] Batista MN, Cuppari L, de Fatima Campos Pedrosa L, Almeida MdG, de Almeida JB, de Medeiros ACQ, Canziani MEF (2006). Effect of end-stage renal disease and diabetes on zinc and copper status. Biol Trace Elem Res.

[B27] Fellah H, Feki M, Souissi M, Ghorbel H, Ben Abdallah T, Massy Z, Hedhili A, Ben Maiz H, Lacour B, Kaabachi N, Mebezza A (2006). Oxidative stress in end stage renal disease: Evidence and association with cardiovascular events in Tunisian patients. Tunis Med.

[B28] Hsieh Y-Y, Shen W-S, Lee L-Y, Wu T-L, Ning H-C, Sun C-F (2006). Long-term changes in trace elements in patients undergoing chronic hemodialysis. Biol Trace Elem Res.

[B29] Kim YS, Park JH, Hong JR, Gil HW, Yang JO, Lee EY, Hong SY (2006). Influence of blood lead concentration on the nerve conduction velocity in patients with end-stage renal disease. J Korean Med Sci.

[B30] Navarro-Alarcon M, Reyes-Perez A, Lopez-Garcia H, Palomares-Bayo M, Olalla-Herrera M, Lopez-Martinez MC (2006). Longitudinal study of serum zinc and copper levels in hemodialysis patients and their relation to biochemical markers. Biol Trace Elem Res.

[B31] Menevse E, Sivrikaya A, Karagozoglu E, Tiftik AM, Turk S (2006). Study of elements, antioxidants and lipid peroxidation in hemodialysis patients. Turk J Med Sci.

[B32] Yilmaz MI, Saglam M, Caglar K, Cakir E, Sonmez A, Ozgurtas T, Aydin A, Eyileten T, Ozcan O, Acikel C, Tasar M, Genctoy G, Erbil K, Vural A, Zoccali C (2006). The determinants of endothelial dysfunction in CKD: oxidative stress and asymmetric dimethylarginine. Am J Kidney Dis.

[B33] Alabdullah H, Bareford D, Braithwaite R, Chipman K (2005). Blood lead levels in iron-deficient and noniron-deficient adults. Clin Lab Haematol.

[B34] Bober J, Kwiatkowska E, Ciechanowski K, Kedzierska K, Dolegowska B, Olszewska M, Kucharska E, Golembiewska E, Chlubek D (2005). Do trace elements modify the activity of erythrocyte sodium-proton exchanger in hemodialyzed patients?. Biol Trace Elem Res.

[B35] Bozalioglu S, Ozkan Y, Turan M, Simsek B (2005). Prevalence of zinc deficiency and immune response in short-term hemodialysis. J Trace Elem Med Biol.

[B36] Cabral PC, Diniz AdS, de Arruda IKG (2005). Vitamin A and zinc status in patients on maintenance haemodialysis. Nephrology.

[B37] Sandhu JS, Narang APS, Bhatia KL, Adlakha J, Khullar D (2005). Trace elements and oxidative stress in chronic renal failure. Trace Elem Electroly.

[B38] Ribeiro RCJ, Sales VSF, Neves FdAR, Draibe S, Brandao-Neto J (2004). Effects of zinc on cell-mediated immunity in chronic hemodialysis patients. Biol Trace Elem Res.

[B39] Yavuz O, Bicik Z, Cinar Y, Guney Y, Guler S (2004). The effect of different dialysis membranes on oxidative stress and selenium status. Clin Chim Acta.

[B40] Zachara BA, Wlodarczyk Z, Masztalerz M, Adamowicz A, Gromadzinska J, Wasowicz W (2004). Selenium concentrations and glutathione peroxidase activities in blood of patients before and after allogenic kidney transplantation. Biol Trace Elem Res.

[B41] Adamowicz A, Trafikowska U, Trafikowska A, Zachara B, Manitius J (2002). Effect of erythropoietin therapy and selenium supplementation on selected antioxidant parameters in blood of uremic patients on long-term hemodialysis. Med Sci Monit.

[B42] Candan F, Gultekin F, Candan F (2002). Effect of vitamin C and zinc on osmotic fragility and lipid peroxidation in zinc-deficient haemodialysis patients. Cell Biochem Funct.

[B43] Pietrzak I, Bladek K, Bulikowski W (2002). Comparison of magnesium and zinc levels in blood in end stage renal disease patients treated by hemodialysis or peritoneal dialysis. Magnes Res.

[B44] Torra M, Rodamilans M, Corbella J (2002). Human exposure to metals: Some factors influencing the metal concentration. Trace Elem Electroly.

[B45] Muniz CS, Fernandez-Martin JL, Marchante-Gayon JM, Garcia Alonso JI, Cannata-Andia JB, Sanz-Medel A (2001). Reference values for trace and ultratrace elements in human serum determined by double-focusing ICP-MS. Biol Trace Elem Res.

[B46] Weissgarten J, Berman S, Bilchinsky R, Modai D, Averbukh Z (2001). Total cell-associated Zn2+ and Cu2+ and proliferative responsiveness of peripheral blood mononuclear cells from patients on chronic hemodialysis. Metabolism.

[B47] Zachara BA, Adamowicz A, Trafikowska U, Trafikowska A, Manitius J, Nartowicz E (2001). Selenium and glutathione levels, and glutathione peroxidase activities in blood components of uremic patients on hemodialysis supplemented with selenium and treated with erythropoietin. J Trace Elem Med Biol.

[B48] Bogye G, Tompos G, Alfthan G (2000). Selenium depletion in hemodialysis patients treated with polysulfone membranes. Nephron.

[B49] Krizek M, Senft V, Motan J (2000). Influence of hemodialysis on selenium blood levels. Sb Lek.

[B50] Lee SH, Huang JW, Hung KY, Leu LJ, Kan YT, Yang CS, Chung Wu D, Huang CL, Chen PY, Chen JS, Chen WY (2000). Trace metals' abnormalities in hemodialysis patients: relationship with medications. Artif Organs.

[B51] Mestek O, Zima T, Suchanek M, Tesar V, Merta M (2000). Determination of copper, selenium and zinc in human blood by inductively coupled mass spectrometry: the sources of uncertainty and variability of results. Sb Lek.

[B52] Roxborough HE, Mercer C, McMaster D, Maxwell AP, Young IS (2000). The ferroxidase activity of caeruloplasmin is reduced in haemodialysis patients. Nephron.

[B53] Hwang SJ, Chang JM, Lee SC, Tsai JH, Lai YH (1999). Short- and long-term uses of calcium acetate do not change hair and serum zinc concentrations in hemodialysis patients. Scand J Clin Lab Invest.

[B54] Bonforte G, Surian M, Dozio B, Scanziani R, Baj A, Colombo S, Toffoletto F (1998). Plasma or whole blood concentrations of trace elements in patients treated by haemodiafiltration with on-line prepared substitution fluid. Nephrol Dial Transplant.

[B55] Chataut CP, Rahman M, Rashid HU, Akhter S, Islam S, Alam M, Ali MS (1998). Serum levels of zinc, copper and magnesium in end stage renal disease subjects and their clinical and biochemical correlations. Bangladesh Renal J.

[B56] Nordio M, Andriani M, Gerotto M, Marchini P, Zambenedetti P, Zatta P (1998). Serum concentration of trace elements during different stages of chronic renal failure. Ital J Miner Elect M.

[B57] Turk S, Bozfakioglu S, Ecder ST, Kahraman T, Gurel N, Erkoc R, Aysuna N, Turkmen A, Bekiroglu N, Ark E (1998). Effects of zinc supplementation on the immune system and on antibody response to multivalent influenza vaccine in hemodialysis patients. Int J Artif Organs.

[B58] Zhang X, Cornelis R, Mees L, Vanholder R, Lameire N (1998). Chemical speciation of arsenic in serum of uraemic patients. Analyst.

[B59] Zima T, Mestek O, Tesar V, Tesarova P, Nemecek K, Zak A, Zeman M (1998). Chromium levels in patients with internal diseases. Biochem Mol Biol Int.

[B60] Hung KY, Ho CY, Kuo YM, Lee SH, Hseih SJ, Yang CS, Peng CJ, Wu DJ, Hung JT, Chen PY (1997). Trace elements burden in geriatric hemodialysis patients: a prospective multicenter collaborative study. Int J Artif Organs.

[B61] Iotova P, Ionova D, Kuleva V, Antonov S, Tzachev K, Nachev C (1997). Zinc in plasma and platelets in patients on regular haemodialysis. Nephrol Dial Transplant.

[B62] Koenig JS, Fischer M, Bulant E, Tiran B, Elmadfa I, Druml W (1997). Antioxidant status in patients on chronic hemodialysis therapy: impact of parenteral selenium supplementation. Wien Klin Wochenschr.

[B63] Bonomini M, Forster S, Manfrini V, De Risio F, Steiner M, Vidovich MI, Klinkmann H, Ivanovich P, Albertazzi A (1996). Geographic factors and plasma selenium in uremia and dialysis. Nephron.

[B64] Emenaker NJ, DiSilvestro RA, Nahman NS, Percival S (1996). Copper-related blood indexes in kidney dialysis patients. Am J Clin Nutr.

[B65] Gunduz Z, Dusunsel R, Kose K, Utas C, Dogan P (1996). The effects of dialyzer reuse on plasma antioxidative mechanisms in patients on regular hemodialysis treatment. Free Radic Biol Med.

[B66] Lin TH, Chen JG, Liaw JM, Juang JG (1996). Trace elements and lipid peroxidation in uremic patients on hemodialysis. Biol Trace Elem Res.

[B67] Marchante-Gayon JM, Sanchez-Uria JE, Sanz-Medel A (1996). Serum and tissue selenium contents related to renal disease and colon cancer as determined by electrothermal atomic absorption spectrometry. J Trace Elem Med Biol.

[B68] Rashid HU, Khan AH, Sarker MH, Akhter S, Khan MF (1996). Effect of haemodialysis on serum zinc, copper and magnesium levels in patients with end stage renal failure. Bangladesh Renal J.

[B69] Romero RA, Salgado O, Rodriguez-Iturbe B, Tahan JE (1996). Blood levels of chromium in diabetic and nondiabetic hemodialysis patients. Transplant Proc.

[B70] Yoshimura S, Suemizu H, Nomoto Y, Sakai H, Katsuoka Y, Kawamura N, Moriuchi T (1996). Plasma glutathione peroxidase deficiency caused by renal dysfunction. Nephron.

[B71] Bonomini M, Manfrini V, Marini A, De Risio F, Niri L, Klinkmann H, Albertazzi A (1995). Hemodialysis with regenerated cellulosic membranes does not reduce plasma selenium levels in chronic uremic patients. Artif Organs.

[B72] Granadillo VA, Salgado O, Barrios Ch L, Romero RA (1995). Distribution of chromium in blood components of diabetics and azotemic patients. Trace Elem Electroly.

[B73] Granadillo VA, Tahan JE, Salgado O, Elejalde LE, Rodriguez-Iturbe B, Romero GB, Romero RA (1995). The influence of the blood levels of lead, aluminum and vanadium upon the arterial hypertension. Clin Chim Acta.

[B74] Cheng PL, Wang Hsu GS (1994). The zinc status in uremic patients with or without hemodialysis. J Chin Nutr Soc.

[B75] Hasanoglu E, Altan N, Sindel S, Ongun CO, Bali M, Altintas E (1994). The relationship between erythrocyte superoxide dismutase activity and plasma levels of some trace elements (Al, Cu, Zn) of dialysis patients. Gen Pharmacol.

[B76] Loughrey CM, Young IS, Lightbody JH, McMaster D, McNamee PT, Trimble ER (1994). Oxidative stress in haemodialysis. QJM.

[B77] Antos M, Jeren-Strujic B, Romic Z, Matanovic B, Gudel-Greguric J (1993). Serum selenium levels in patients on hemodialysis. Trace Elem Med.

[B78] Colleoni N, Arrigo G, Gandini E, Corigliano C, D'Amico G (1993). Blood lead in hemodialysis patients. Am J Nephrol.

[B79] De Kimpe J, Cornelis R, Mees L, Van Lierde S, Vanholder R (1993). More than tenfold increase of arsenic in serum and packed cells of chronic hemodialysis patients. Am J Nephrol.

[B80] Girelli D, Olivieri O, Stanzial AM, Azzini M, Lupo A, Bernich P, Menini C, Gammaro L, Corrocher R (1993). Low platelet glutathione peroxidase activity and serum selenium concentration in patients with chronic renal failure: relations to dialysis treatments, diet and cardiovascular complications. Clin Sci (Colch).

[B81] Holtkamp W, Brodersen HP, Stollberg T, Thiery J, Falkner C (1993). Zinc supplementation stimulates tetanus antibody formation and soluble interleukin-2 receptor levels in chronic hemodialysis patients. Clin Investig.

[B82] Hosokawa S, Yoshida O (1993). Effects of erythropoietin on trace elements in patients with chronic renal failure undergoing hemodialysis. Nephron.

[B83] Mayer DR, Kosmus W, Pogglitsch H, Mayer D, Beyer W (1993). Essential trace elements in humans. Serum arsenic concentrations in hemodialysis patients in comparison to healthy controls. Biol Trace Elem Res.

[B84] Shu KH, Lu YS, Chen CH, Chen DC, Lee SH, Lian JD (1993). Lymphocyte proliferation in uremic patients: correlation with zinc status. J Formos Med Assoc.

[B85] Mihailovic M, Lindberg P, Jovanovic I, Antic D (1992). Selenium status of patients with Balkan endemic nephropathy. Biol Trace Elem Res.

[B86] Milly K, Wit L, Diskin C, Tulley R (1992). Selenium in renal failure patients. Nephron.

[B87] Navarro JA, Granadillo VA, Salgado O, Rodriguez-Iturbe B, Garcia R, Delling G, Romero RA (1992). Bone metal content in patients with chronic renal failure. Clin Chim Acta.

[B88] Turan B, Delilbasi E, Dalay N, Sert S, Afrasyap L, Sayal A (1992). Serum selenium and glutathione-peroxidase activities and their interaction with toxic metals in dialysis and renal transplantation patients. Biol Trace Elem Res.

[B89] Kouw PM, Konings CH, de Vries PM, Meulen J van der, Oe PL (1991). Effects of zinc supplementation on zinc status and immunity in haemodialysis patients. J Trace Elem Elect H.

[B90] Richard MJ, Arnaud J, Jurkovitz C, Hachache T, Meftahi H, Laporte F, Foret M, Favier A, Cordonnier D (1991). Trace elements and lipid peroxidation abnormalities in patients with chronic renal failure. Nephron.

[B91] Clyne N, Lins LE, Pehrsson SK (1990). Serum cobalt in relation to residual renal function in uremic patients before and after renal transplantation. Trace Elem Med.

[B92] Kostakopoulos A, Kotsalos A, Alexopoulos J, Sofras F, Deliveliotis C, Kallistratos G (1990). Serum selenium levels in healthy adults and its changes in chronic renal failure. Int Urol Nephrol.

[B93] Romero RA, Navarro JA, Rodriguez-Iturbe B, Parra OE, Granadillo VA (1990). Distribution of trace metals in blood components of patients with chronic renal failure undergoing periodical hemodialysis treatment. Trace Elem Med.

[B94] Togni E, Travaglini P, Beretta C, Berardinelli L, Vegeto A, Mocchegiani E, Fabris N, Egidi F, Ponticelli C, Faglia G (1990). Prolactin, thymulin and zinc in chronic hemodialysis: effect of renal transplant. J Endocrinol Invest.

[B95] Tsukamoto Y, Saka S, Kumano K, Iwanami S, Ishida O, Marumo F (1990). Abnormal accumulation of vanadium in patients on chronic hemodialysis therapy. Nephron.

[B96] Agenet C, Brugere CC, Reynier JP (1989). Plasma and intra-erythrocytic concentrations of copper and zinc in uremic patients treated by periodic hemodialysis. Ann Biol Clin (Paris).

[B97] Hachache T, Meftahi H, Foret M, Kuentz F, Milongo R, Christollet M, Cordonnier DJ, Arnaud J, Favier A (1989). Short- (1 session) and long-term (6 months) course of the serum level of zinc in 33 hemodialysis patients. Nephrologie.

[B98] Hopfer SM, Fay WP, Sunderman FW (1989). Serum nickel concentrations in hemodialysis patients with environmental exposure. Ann Clin Lab Sci.

[B99] Ishida O, Kihira K, Tsukamoto Y, Marumo F (1989). Improved determination of vanadium in biological fluids by electrothermal atomic absorption spectrometry. Clin Chem.

[B100] Mahajan SK, Bowersox EM, Rye DL, Abu-Hamdan DK, Prasad AS, McDonald FD, Biersack KL (1989). Factors underlying abnormal zinc metabolism in uremia. Kidney Int Suppl.

[B101] Navarro J, Parra O, Garcia R, Rodriguez-Iturbe B, Granadillo V, Rubio P, Romero R (1989). Trace metal levels during hemodialysis in patients with chronic renal failure. Trace Elem Med.

[B102] Nixon DE, Moyer TP, Squillace DP, McCarthy JT (1989). Determination of serum nickel by graphite furnace atomic absorption spectrometry with Zeeman-effect background correction: values in a normal population and a population undergoing dialysis. Analyst.

[B103] Saint-Georges MD, Bonnefont DJ, Bourely BA, Jaudon MC, Cereze P, Chaumeil P, Gard C, D'Auzac CL (1989). Correction of selenium deficiency in hemodialyzed patients. Kidney Int Suppl.

[B104] Sampson B, Curtis JR, Davies S (1989). Survey of blood lead and plasma aluminium concentrations in patients of a renal unit. Nephrol Dial Transplant.

[B105] Travaglini P, Moriondo P, Togni E, Venegoni P, Bochicchio D, Conti A, Ambroso G, Ponticelli C, Mocchegiani E, Fabris N (1989). Effect of oral zinc administration on prolactin and thymulin circulating levels in patients with chronic renal failure. J Clin Endocrinol Metab.

[B106] Abu-Hamdan DK, Mahajan SK, Migdal S, Prasad AS, McDonald FD (1988). Zinc tolerance test in uremia: effect of calcitriol supplementation. J Am Coll Nutr.

[B107] Foote JW, Hinks LJ (1988). Zinc absorption in haemodialysis patients [see comment]. Ann Clin Biochem.

[B108] Kuroda M, Imura T, Morikawa K, Hasegawa T (1988). Decreased serum levels of selenium and glutathione peroxidase activity associated with aging, malignancy and chronic hemodialysis. Trace Elem Med.

[B109] Mendes V, Romao Junior JE, Kirschbaum E, Andriollo A, Marcondes M, Sabbaga E (1988). Serum zinc in patients undergoing chronic hemodialysis. Rev Hosp Clin Fac Med Sao Paulo.

[B110] Sondheimer JH, Mahajan SK, Rye DL, Abu-Hamdan DK, Migdal SD, Prasad AS, McDonald FD (1988). Elevated plasma copper in chronic renal failure. Am J Clin Nutr.

[B111] Chen HJ, Lai YH, Lin SM, Tsai JH (1987). Taste acuity and zinc in uremic patients undergoing long-term hemodialysis. Taiwan I Hsueh Hui Tsa Chih.

[B112] Dworkin B, Weseley S, Rosenthal WS, Schwartz EM, Weiss L (1987). Diminished blood selenium levels in renal failure patients on dialysis: correlations with nutritional status. Am J Med Sci.

[B113] Foote JW, Hinks LJ (1987). Reduced leucocyte zinc and albumin-bound zinc in blood of haemodialysis patients. Ann Clin Biochem.

[B114] Foote JW, Hinks LJ, Lloyd B (1987). Reduced plasma and white blood cell selenium levels in haemodialysis patients. Clin Chim Acta.

[B115] Hosokawa S, Nishitani H, Umemura K, Tomoyoshi T, Sawanishi K, Yoshida O (1987). Serum and corpuscular nickel and zinc in chronic hemodialysis patients. Nephron.

[B116] Ruiz Alcantarilla P, Lozano Diaz A, Gomez Rodriguez F (1987). Plasma zinc levels, nutritional status and immune response in chronic renal insufficiency. Rev Clin Esp.

[B117] Sanada S, Kuze M, Yoshida O (1987). Beneficial effect of zinc supplementation on pruritus in hemodialysis patients with special reference to changes in serum histamine levels. Hinyokika Kiyo.

[B118] Shu KH, Lian JD, Yang YF, Lu YS, Cheng CH, Chen DL (1987). Plasma zinc level in hemodialysis, continuous ambulatory peritoneal dialysis and renal transplantation. Taiwan I Hsueh Hui Tsa Chih.

[B119] Abu-Hamdan DK, Mahajan SK, Migdal SD, Prasad AS, McDonald FD (1986). Zinc tolerance test in uremia. Effect of ferrous sulfate and aluminum hydroxide. Ann Intern Med.

[B120] Chen HJ, Lai YH, Lin SM, Tsai JH (1986). Serum copper and effect of dialysis on it in uremic patients. Taiwan I Hsueh Hui Tsa Chih.

[B121] Hosokawa S, Koike T, Kawaji A, Nishitani H, Nishio T, Tomoyoshi T, Sawanishi K, Yoshida O (1986). Zinc transport during hemodialysis. Artif Organs.

[B122] Mauras Y, Ang KS, Simon P, Tessier B, Cartier F, Allain P (1986). Increase in blood plasma levels of boron and strontium in hemodialyzed patients. Clin Chim Acta.

[B123] Drazniowsky M, Parkinson IS, Ward MK, Channon SM, Kerr DN (1985). Raised serum nickel concentrations in chronic renal failure. Proc Eur Dial Transplant Assoc Eur Ren Assoc.

[B124] Hosokawa S, Nishitani H, Umemura K, Nishio T, Tomoyoshi T, Sawanishi K, Yoshida O (1985). Relationship between haemodialysis anaemia and copper and zinc. Int Urol Nephrol.

[B125] Hosokawa S, Nishitani H, Tomita K, Tomoyoshi T, Nishio T, Sawanishi K, Yoshida O (1985). Serum copper concentration changes in chronic hemodialyzed patients. Uremia Invest.

[B126] Kallistratos G, Evangelou A, Seferiadis K, Vezyraki P, Barboutis K (1985). Selenium and haemodialysis: serum selenium levels in healthy persons, non-cancer and cancer patients with chronic renal failure. Nephron.

[B127] Wills MR, Brown CS, Bertholf RL, Ross R, Savory J (1985). Serum and lymphocyte, aluminum and nickel in chronic renal failure. Clin Chim Acta.

[B128] Minami T, Samukawa K, Adachi K, Okazaki Y (1984). Change of serum chromium level in chronic hemodialysis patients. Yakugaku Zasshi.

[B129] Piechota W, Dobrucki T, Symonowicz N, Wadowska E, Murkowska E (1983). Zinc in patients with chronic renal failure. Int Urol Nephrol.

[B130] Thomson NM, Stevens BJ, Humphery TJ, Atkins RC (1983). Comparison of trace elements in peritoneal dialysis, hemodialysis, and uremia. Kidney Int.

[B131] Mahajan SK, Prasad AS, Rabbani P, Briggs WA, McDonald FD (1982). Zinc deficiency: a reversible complication of uremia. Am J Clin Nutr.

[B132] Okuyama S, Mishina H, Hasegawa K, Nakano N, Ise K (1982). Probable atherogenic role of zinc and copper as studied in chronic hemodialysis patients. Tohoku J Exp Med.

[B133] Temes-Montes XL, Picaporte MA, Herrero E, Otero A, Sanchez-Guisande D, Sanchez-Sicilia L (1982). Zinc in patients with chronic renal insufficiency subjected to periodic maintenance hemodialysis. Rev Clin Esp.

[B134] Paniagua-Sierra JR, Perez-Lopez A, Diaz-Bensussen S, Solis-Alpuche L, Saavedra-Guatemala H, Exaire-Murad JE (1981). Zinc and copper concentration in plasma and erythrocytes of patients with chronic renal failure. Arch Invest Med (Mex).

[B135] Schiffl HH, Binswanger U (1980). Human urinary fluoride excretion as influenced by renal functional impairment. Nephron.

[B136] Tsukamoto Y, Iwanami S, Marumo F (1980). Disturbances of trace element concentrations in plasma of patients with chronic renal failure. Nephron.

[B137] Cornelis R, Mees L, Ringoirs S, Hoste J (1979). Serum and red blood cell Zn, Se, Cs and Rb in dialysis patients. Miner Electrolyte Metab.

[B138] Mahajan SK, Prasad AS, Rabbani P, Briggs WA, McDonald FD (1979). Zinc metabolism in uremia. J Lab Clin Med.

[B139] Marumo F, Tsukamoto Y, Iwanami S, Kishimoto T, Yamagami S (1979). Effects of hemofiltration and hemodialysis on contents of trace elements in hair, nails and plasma of patients with chronic renal failure. Proc Clin Dial Transplant Forum.

[B140] Mountokalakis T, Dakanalis D, Boukis D, Virvidakis K, Voudiklari S, Koutselinis A (1979). Hair zinc compared with plasma zinc in uremic patients before and during regular hemodialysis. Clin Nephrol.

[B141] Zumkley H, Bertram HP, Lison A, Knoll O, Losse H (1979). Aluminum, zinc and copper concentrations in plasma in chronic renal insufficiency. Clin Nephrol.

[B142] Mahajan SK, Gardiner WH, Abbasi AA, Briggs WA, Prasad AS, McDonald FD (1978). Abnormal plasma and erythrocyte zinc distribution in uremia. Trans Am Soc Artif Intern Organs.

[B143] Willden EG, Hyne BE (1974). Blood and urinary cadmium in chronic renal failure. Nephron.

[B144] Rudolph H, Alfrey AC, Smythe WR (1973). Muscle and serum trace element profile in uremia. Trans Am Soc Artif Intern Organs.

[B145] Rose GA, Willden EG (1972). Whole blood, red cell and plasma total and ultrafiltrable zinc levels in normal subjects and patients with chronic renal failure with and without haemodialysis. Br J Urol.

[B146] Barbour BH, Bischel M, Abrams DE (1971). Copper accumulation in patients undergoing chronic hemodialysis. The role of cuprophan. Nephron.

[B147] Mahler DJ, Walsh JR, Haynie GD (1971). Magnesium, zinc, and copper in dialysis patients. Am J Clin Pathol.

[B148] Mansouri K, Halsted JA, Gombos EA (1970). Zinc, copper, magnesium and calcium in dialyzed and nondialyzed uremic patients. Arch Intern Med.

[B149] Zazgornik J, Kotzaurek R, Schmidt P (1971). Plasma zinc concentration in uremic patients during hemodialysis. Klin Wochenschr.

[B150] Rink L, Gabriel P (2000). Zinc and the immune system. Proc Nutr Soc.

[B151] Shankar AH, Prasad AS (1998). Zinc and immune function: the biological basis of altered resistance to infection. Am J Clin Nutr.

[B152] Collins AJ, Kasiske B, Herzog C, Chavers B, Foley R, Gilbertson D, Grimm R, Liu J, Louis T, Manning W, Matas A, McBean M, Murray A, St Peter W, Xue J, Fan Q, Guo H, Li S, Roberts T, Snyder J, Solid C, Wang C, Weinhandl E, Arko C, Chen SC, Dalleska F, Daniels F, Dunning S, Ebben J, Frazier E (2005). Excerpts from the United States Renal Data System 2004 annual data report: atlas of end-stage renal disease in the United States. Am J Kidney Dis.

[B153] Oliver MJ, Rothwell DM, Fung K, Hux JE, Lok CE (2004). Late creation of vascular access for hemodialysis and increased risk of sepsis. J Am Soc Nephrol.

[B154] Allon M (2004). Dialysis catheter-related bacteremia: treatment and prophylaxis. Am J Kidney Dis.

[B155] Mokrzycki MH, Zhang M, Cohen H, Golestaneh L, Laut JM, Rosenberg SO (2006). Tunnelled haemodialysis catheter bacteraemia: risk factors for bacteraemia recurrence, infectious complications and mortality. Nephrol Dial Transplant.

[B156] O'Seaghdha CM, Foley RN (2005). Septicemia, access, cardiovascular disease, and death in dialysis patients. Perit Dial Int.

[B157] Ishani A, Collins AJ, Herzog CA, Foley RN (2005). Septicemia, access and cardiovascular disease in dialysis patients: the USRDS Wave 2 study. Kidney Int.

[B158] Halliwell B (1982). Ascorbic acid, iron overload, and desferrioxamine [letter]. Br Med J (Clin Res Ed).

[B159] Markovits PM, Sankey AW, James DK, McCabe R, Mahomed K, Golding J (1990). Zinc taste test and postnatal depression. Br J Psychiatry.

[B160] Ortega RM, Requejo AM, Andres P, Lopez-Sobaler AM, Quintas ME, Redondo MR, Navia B, Rivas T (1997). Dietary intake and cognitive function in a group of elderly people. Am J Clin Nutr.

[B161] Boosalis MG (2008). The role of selenium in chronic disease. Nutr Clin Pract.

[B162] Nawrot TS, Staessen JA, Roels HA, Den Hond E, Thijs L, Fagard RH, Dominiczak AF, Struijker-Boudier HA (2007). Blood pressure and blood selenium: a cross-sectional and longitudinal population study. Eur Heart J.

[B163] Witte KK, Clark AL, Cleland JG (2001). Chronic heart failure and micronutrients. J Am Coll Cardiol.

[B164] Bhattacharya SK, Ahokas RA, Carbone LD, Newman KP, Gerling IC, Sun Y, Weber KT (2006). Macro- and micronutrients in African-Americans with heart failure. Heart Fail Rev.

[B165] Flores-Mateo G, Navas-Acien A, Pastor-Barriuso R, Guallar E (2006). Selenium and coronary heart disease: a meta-analysis. Am J Clin Nutr.

[B166] Hampel G, Schaller KH, Rosenmuller M, Oefele C (1985). Selenium-deficiency as contributing factor to anemia and thrombocytopenia in dialysis patients. Life Support Syst.

[B167] Girelli D, Olivieri O, Stanzial AM, Azzini M, Lupo A, Bernich P, Menini C, Gammaro L, Corrocher R (1993). Low platelet glutathione peroxidase activity and serum selenium concentration in patients with chronic renal failure: relations to dialysis treatments, diet and cardiovascular complications. Clin Sci (Lond).

[B168] Rayman MP, Rayman MP (2002). The argument for increasing selenium intake. Proc Nutr Soc.

[B169] Klotz LO, Kroncke KD, Buchczyk DP, Sies H (2003). Role of copper, zinc, selenium and tellurium in the cellular defense against oxidative and nitrosative stress. J Nutr.

[B170] Stenvinkel P (2003). Interactions between inflammation, oxidative stress, and endothelial dysfunction in end-stage renal disease. J Ren Nutr.

[B171] Ward RA, McLeish KR (2003). Oxidant stress in hemodialysis patients: what are the determining factors?. Artif Organs.

[B172] Hays GL, Bullock Q, Lazzari EP, Puente ES (1992). Salivary pH while dissolving vitamin C-containing tablets. Am J Dent.

[B173] Bleecker ML, Lindgren KN, Ford DP (1997). Differential contribution of current and cumulative indices of lead dose to neuropsychological performance by age. Neurology.

[B174] Schwartz BS, Bolla KI, Stewart W, Ford DP, Agnew J, Frumkin H (1993). Decrements in neurobehavioral performance associated with mixed exposure to organic and inorganic lead. Am J Epidemiol.

[B175] Nawrot TS, Thijs L, Den Hond EM, Roels HA, Staessen JA (2002). An epidemiological re-appraisal of the association between blood pressure and blood lead: a meta-analysis. J Hum Hypertens.

[B176] Schwartz J (1995). Lead, blood pressure, and cardiovascular disease in men. Arch Environ Health.

[B177] Kim R, Rotnitsky A, Sparrow D, Weiss S, Wager C, Hu H (1996). A longitudinal study of low-level lead exposure and impairment of renal function. The Normative Aging Study. JAMA.

[B178] Malcolm D, Barnett HA (1982). A mortality study of lead workers 1925–76. Brit J Ind Med.

[B179] Pirkle JL, Schwartz J, Landis JR, Harlan WR (1985). The relationship between blood lead levels and blood pressure and its cardiovascular risk implications. Am J Epidemiol.

[B180] Huang C, Ke Q, Costa M, Shi X (2004). Molecular mechanisms of arsenic carcinogenesis. Mol Cell Biochem.

[B181] Kitchin KT, Ahmad S (2003). Oxidative stress as a possible mode of action for arsenic carcinogenesis. Toxicol Lett.

[B182] Zhao CQ, Young MR, Diwan BA, Coogan TP, Waalkes MP (1997). Association of arsenic-induced malignant transformation with DNA hypomethylation and aberrant gene expression. Proc Natl Acad Sci USA.

[B183] Hei TK, Liu SX, Waldren C (1998). Mutagenicity of arsenic in mammalian cells: role of reactive oxygen species. Proc Natl Acad Sci USA.

[B184] Tseng CH (2005). Blackfoot disease and arsenic: a never-ending story. J Environ Sci Health C.

[B185] Yu HS, Lee CH, Chen GS (2002). Peripheral vascular diseases resulting from chronic arsenical poisoning. J Dermatol.

[B186] Biswas S, Talukder G, Sharma A (1999). Prevention of cytotoxic effects of arsenic by short-term dietary supplementation with selenium in mice *in vivo*. Mutat Res.

[B187] Schoen A, Beck B, Sharma R, Dube E (2004). Arsenic toxicity at low doses: epidemiological and mode of action considerations. Toxicol Appl Pharmacol.

[B188] (1995). Medicare program; standards for quality of water used in dialysis and revised guidelines on reuse of hemodialysis filters for end-stage renal disease (ESRD) patients – HCFA. Final rule. Fed Regist.

[B189] Wills MR, Savory J (1989). Aluminum and chronic renal failure: sources, absorption, transport, and toxicity. Crit Rev Clin Lab Sci.

[B190] Kerr DN, Ward MK, Ellis HA, Simpson W, Parkinson IS (1992). Aluminium intoxication in renal disease. Ciba Found Symp.

[B191] Alfrey AC (1978). Dialysis encephalopathy syndrome. Annu Rev Med.

[B192] Altmann P, Al-Salihi F, Butter K, Cutler P, Blair J, Leeming R, Cunningham J, Marsh F (1987). Serum aluminum levels and erythrocyte dihydropteridine reductase activity in patients on hemodialysis. N Engl J Med.

[B193] Alfrey AC, LeGendre GR, Kaehny WD (1976). The dialysis encephalopathy syndrome. Possible aluminum intoxication. N Engl J Med.

[B194] McIvor M, Baltazar RF, Beltran J, Mower MM, Wenk R, Lustgarten J, Salomon J (1983). Hyperkalemia and cardiac arrest from fluoride exposure during hemodialysis. Am J Cardiol.

[B195] Arnow PM, Bland LA, Garcia-Houchins S, Fridkin S, Fellner SK (1994). An outbreak of fatal fluoride intoxication in a long-term hemodialysis unit. Ann Intern Med.

[B196] (1980). Fatal fluoride intoxication in a dialysis unit. J Public Health Dent.

[B197] Chevalier CA, Liepa G, Murphy MD, Suneson J, Vanbeber AD, Gorman MA, Cochran C (2002). The effects of zinc supplementation on serum zinc and cholesterol concentrations in hemodialysis patients. J Ren Nutr.

[B198] Candan F, Gultekin F, Candan F (2002). Effect of vitamin C and zinc on osmotic fragility and lipid peroxidation in zinc-deficient haemodialysis patients. Cell Biochem Funct.

[B199] Zachara BA, Adamowicz A, Trafikowska U, Trafikowska A, Manitius J, Nartowicz E (2001). Selenium and glutathione levels, and glutathione peroxidase activities in blood components of uremic patients on hemodialysis supplemented with selenium and treated with erythropoietin. J Trace Elem Med Biol.

[B200] Temple KA, Smith AM, Cockram DB (2000). Selenate-supplemented nutritional formula increases plasma selenium in hemodialysis patients. J Ren Nutr.

[B201] McElroy BH, Miller SP (2003). An open-label, single-center, phase IV clinical study of the effectiveness of zinc gluconate glycine lozenges (Cold-Eeze) in reducing the duration and symptoms of the common cold in school-aged subjects. Am J Ther.

[B202] Bobat R, Coovadia H, Stephen C, Naidoo KL, McKerrow N, Black RE, Moss WJ (2005). Safety and efficacy of zinc supplementation for children with HIV-1 infection in South Africa: a randomised double-blind placebo-controlled trial. Lancet.

[B203] Girodon F, Galan P, Monget AL, Boutron-Ruault MC, Brunet-Lecomte P, Preziosi P, Arnaud J, Manuguerra JC, Herchberg S (1999). Impact of trace elements and vitamin supplementation on immunity and infections in institutionalized elderly patients: a randomized controlled trial. MIN. VIT. AOX. geriatric network. Arch Intern Med.

[B204] Field CJ, Johnson IR, Schley PD (2002). Nutrients and their role in host resistance to infection. J Leukoc Biol.

[B205] Aggarwal R, Sentz J, Miller MA (2007). Role of zinc administration in prevention of childhood diarrhea and respiratory illnesses: a meta-analysis. Pediatrics.

[B206] Bhutta ZA, Black RE, Brown KH, Gardner JM, Gore S, Hidayat A, Khatun F, Martorell R, Ninh NX, Penny ME, Rosado JL, Roy SK, Ruel M, Sazawal S, Shankar A (1999). Prevention of diarrhea and pneumonia by zinc supplementation in children in developing countries: pooled analysis of randomized controlled trials. Zinc Investigators' Collaborative Group. J Pediatr.

[B207] Baqui AH, Black RE, El Arifeen S, Yunus M, Chakraborty J, Ahmed S, Vaughan JP (2002). Effect of zinc supplementation started during diarrhoea on morbidity and mortality in Bangladeshi children: community randomised trial. BMJ.

[B208] Muller O, Becher H, van Zweeden AB, Ye Y, Diallo DA, Konate AT, Gbangou A, Kouyate B, Garenne M (2001). Effect of zinc supplementation on malaria and other causes of morbidity in west African children: randomised double blind placebo controlled trial. BMJ.

[B209] Sazawal S, Black RE, Menon VP, Dinghra P, Caulfield LE, Dhingra U, Bagati A (2001). Zinc supplementation in infants born small for gestational age reduces mortality: a prospective, randomized, controlled trial. Pediatrics.

[B210] Patel AB, Dhande LA, Rawat MS (2005). Therapeutic evaluation of zinc and copper supplementation in acute diarrhea in children: double blind randomized trial. Indian Pediatr.

[B211] Brooks WA, Santosham M, Naheed A, Goswami D, Wahed MA, Diener-West M, Faruque AS, Black RE (2005). Effect of weekly zinc supplements on incidence of pneumonia and diarrhoea in children younger than 2 years in an urban, low-income population in Bangladesh: randomised controlled trial. Lancet.

[B212] Skarupskiene I, Kuzminskis V, Abdrachmanovas O, Ryselis S, Smalinskiene A (2005). Cinko ir aliuminio kiekio hemodializuojamu ligoniu kraujyje itaka infekciniu komplikaciju dazniui. Medicina (Kaunas).

[B213] Hu H, Shih R, Rothenberg S, Schwartz BS (2007). The epidemiology of lead toxicity in adults: measuring dose and consideration of other methodologic issues. Environ Health Perspect.

