# Supplementary material for: Trace elements in hemodialysis patients: a systematic review and meta-analysis
Source: BMC Med. 2009 May 19;7:25. doi: 10.1186/1741-7015-7-25 (PMC2698829; doi:10.1186/1741-7015-7-25)
Supplement: Additional file 1 — Appendix. Literature search strategies. [file 1741-7015-7-25-S1.doc]

| **Appendix Table 1. Description of included studies** | | | | | | | | |
| --- | --- | --- | --- | --- | --- | --- | --- | --- |
| Author Year Country | Study design  Element | Sample Technique | Hemodialysis Participants | | | | Healthy Controls | |
|  |  |  | N  Age* y, Male % | Cause of ESRD Co-morbidities | Months on HD Median Range | Membrane Flux | Control Co-morbidities | N Age* y, Male % |
| Bober 2007[24]  Poland | CS  Se | P  FM | 43  60, - | -  - | 9  - | PS  low/high | -  - | 21  - |
| Zagrodzki 2007[25]  Poland | CS  Se | P  AAS | 69  55, 64 | DM, GN, PCKD, Other  - | 54  15-278 | PS  low | -  - | 40  40, 50 |
| Batista 2006[26] Brazil | PC  Cu, Zn | Pl AAS | 63 57, 49 | - DM, NDM | 36 - | Cellulose - | Staff - | 20 53, 40 |
| Fellah 2006[27]  France | CS  Cu, Se, Zn | Pl  AAS | 50  41, 50 | -  - | >2 y  - | PS  - | -  - | 31  48, 48 |
| Hsieh 2006[28] Taiwan | CS  Cu, Mn, Ni, Se, Zn | S ICP/MS | 77 - | - - | 55 - | - - | Non-renal patients - | 51 - |
| Kim 2006[29] Korea | CS  Pb | WB AAS | 68 50, 46 | - - | 3.6 y 0-12 y | CA - | Non-renal patients - | 198 43, 74 |
| Navarro-Alarcon 2006[30]  Spain | PC  Cu, Zn | S  FLAAS | 48  53, 67 | GN, Other  - | -  - | PS  high | Geographic volunteers  - | 52  50, 46 |
| Menevse 2006[31] Turkey | CS  Se | S ICP/ES | 47 50, 28 | - - | 2-16 y | PS - | Volunteers - | 23 40, 70 |
| Yilmaz 2006[32] Turkey | CS  Cu, Se | Pl AAS | 159 44, 54 | GN, HTN, Other - | - >1 y | - - | - - | 30 44, 50 |
| Alabdullah 2005[33] United Kingdom | CS  Pb | WB ETAAS | 78 - | - - | - - | - - | Blood donors - | 30 - |
| Bober 2005[34] Poland | CS  Cu, Se, Zn | Pl  AAS | 21 - | GN, Other - | - - | PS - | - - | 21 - |
| Bozalioglu 2005[35] Turkey | CS  Zn | Pl FAAS | 72 48, 57 | - - | - 48-50 | C - | - - | 52 37, 52 |
| Cabral 2005[36] Brazil | Case series  Zn | S AAS | 55 48, 52 | - - | 43 - | - - | Volunteers - | 28 46, 57 |
| Sandhu 2005[37] India | CS  Cu, Se, Zn | S AAS | 10 36, 90 | - - | - - | - - | - - | 10 39, 70 |
| Ribeiro 2004[38] Brazil | PC  Zn | S AAS | 12 41, - | GN, HTN, Other - | 18 mean - | - - | Staff - | 13 - |
| Yavuz 2004[39] Turkey | RCT  Se | S AAS | 40 51, 45 | HTN, DM, PCKD, Other - | 41 10-59 | H - | Volunteers - | 20 51, 40 |
| Zachara 2004[40] Poland | PC  Se | WB, Pl FM | 30 48, 50 | - - | - - | - - | Volunteers - | 20 43, 45 |
| Adamowicz 2002[41] Poland | PC  Se | WB, Pl - | 11 37, 61 | - - | - - | - - | - - | 18 41, 55 |
| Candan 2002[42] Turkey | PC  Zn | Pl AAS | 34 46, 53 | - - | - - | - - | Volunteers - | 16 47, 38 |
| Pietrzak 2002[43] Poland | CS  Cr, Zn | Pl AAS | 15 41, 36 | GN, PCKD, Other - | 49 - | C - | - - | 45 41, 82 |
| Torra 2002[44] Spain | CS  Cr, Pb, Mn | WB GFAAS | 105 range 20-77, 51 | - - | - - | - - | Geographic volunteers - | 200 range 22-79, 55 |
| Muniz 2001[45] Spain | CS  Cu, Mo, Zn | S ICP/MS | 14 -, 79 | - - | - - | - - | Blood donors - | 59 40, 69 |
| Weissgarten 2001[46] Israel | CS  Cu, Zn | Pl AAS | 10 74, 70 | GN, HTN, PCKD, Other - | 4 1-7 | - - | - - | 10 74, 70 |
| Zachara 2001[47] Poland | PC  Se | WB, Pl FM | 58 40, - | - - | 42 3-102 | - - | - - | 25 38, - |
| Bogye 2000[48] Hungary | Case control  Se | S ETAAS | 28 59, - | - - | - - | PS - | Staff, Blood donors - | 32 59, - |
| Krizek 2000[49] Czech Republic | PC  Se | WB AAS | 95 61, 45 | GN, PCKD, DM, Other - | 32 1-290 | C, H, PS  - | Volunteers - | 20 59, 50 |
| Lee 2000[50] Taiwan | CS  Cd, Cu, Pb, Hg, Zn | WB, Pl AAS | 456 53, - | - - | - ≥3 | Synthetic and semi-synthetic - | Non-renal patients  - | 152 54, - |
| Mestek 2000[51] Czech Republic | PC  Cu, Se, Zn | WB, Pl ICP/MS | 17 - | GN, PCKD, Other  - | - - | - - | Blood donors - | 12 - |
| Roxborough 2000[52] Ireland | CS  Cu | Pl AAS | 83 48, 49 | GN, DM, PCKD, Other - | - - | PS - | - - | 52 51, 52 |
| Hwang 1999[53] Taiwan | PC  Zn | S AAS | 10 50, - | - - | 56 - | - - | Staff - | 16 41, - |
| Bonforte 1998[54] Italy | PC  Cd, Cr, Cu, Pb, Se, Zn | WB, Pl AAS | 20 - | - - | - - | - - | - - | 490 - |
| Chataut 1998[55]  Bangladesh | CS  Cu, Zn | S  AAS | 20  - | -  - | -  >6 | -  - | -  - | 40  -, 53 |
| Nordio 1998[56]  Italy | CS  Cu, Zn | Pl  ICP/MS | 10  - | -  - | -  - | -  - | -  - | 10  44, 50 |
| Turk 1998[57] Turkey | PC  Zn | S AAS | 26 42, 38 | - - | 66 >10 | - - | - - | 11 39, 55 |
| Zhang 1998[58] Belgium | CS  As | S AAS | 18 - | - - | - - | - - | Reference - | 23 - |
| Zima 1998 A[2] Czech Republic | CS  Cu, Se, Zn | WB, Pl ICP/MS | 36 57, 19 | - - | - - | C, H low | Blood donors - | 42 40, 24 |
| Zima 1998 B[59] Czech Republic | CS  Cr | S AAS & ETAAS | 19 54, 42 | - - | - - | C, H low | Blood donors - | 19 41, 42 |
| Hung 1997[60] Taiwan | PC  Cd, Cu, Pb, Hg, Zn | WB, Pl AAS | 151 68, 65 | - - | - ≥6 | Cellulose - | Non-renal patients - | 112 68, 58 |
| Iotova 1997[61] Bulgaria | CS  Zn | Pl FLAAS & ETAAS | 65 51, - | - - | 63 - | - - | - - | 42 49, - |
| Koenig 1997[62] Austria | PC  Se | Pl HGAAS | 12 58, 50 | GN, DM, Other  - | 46 5-159 | - - | - - | 17 - |
| Bonomini 1996[63] Italy/Germany | CS  Se | Pl AAS | 47 50, 57 | - - | 51 - | C, CA/PS, PAN, PA low/high | Geographic volunteers - | 144 42, 52 |
| Emenaker 1996[64] USA | CS  Cu | Pl AAS | 23 50, - | - - | - - | CA, cellulose esters, PS - | - - | 23 Matched, - |
| Gunduz 1996[65] Turkey | CS  Cu | Pl AAS | 12 32, 42 | GN, PCKD, Other - | 21 10-46 | C - | - - | 12 29, 58 |
| Lin 1996[66] Taiwan | CS  Cu, Pb, Mn, Se, Zn | Pl FLAAS | 26 57, 15 | - - | 56 6-154 | - - | Reference - | 25 range 21-50, 10 |
| Marchante-Gayon 1996[67] Spain | CS  Se | S AAS | 22 >18, - | - - | - - | - - | Geographic volunteers - | 22 >18, - |
| Rashid 1996[68] Bangladesh | CS  Cu, Zn | S FLAAS | 45 38, 80 | - - | - - | - - | Staff, Volunteers - | 60 33, 67 |
| Romero 1996[69] Venezuela | CS  Cr | WB, S, Pl ETAAS | 55 range 27-63, 51 | -  DM, NDM | 69 - | - - | - - | 30 range 24-51, 50 |
| Usuda 1996[16] Japan | CS  B | S ICP/ES | 17 61, 41 | - - | - - | PV - | Reference - | 467 Matched, 32 |
| Yoshimura 1996[70]  Japan | CS  Se | Pl AAS | 20 64, 45 | - - | - 1-144 | CA - | Geographic volunteers - | 118 50, 47 |
| Bonomini 1995[71]  Italy | PC  Se | Pl AAS | 20 59, 100 | - - | 63 mean - | C low | - - | 28 Matched, 100 |
| Granadillo 1995 A[72] Venezuela | CS  Cr | WB, S, Pl ETAAS | 30 33, 43 | - - | 13 2 wks-5 y | - - | Volunteers - | 30 36, 50 |
| Granadillo 1995 B[73] Venezuela | CS  Pb, V | WB, Pl ETAAS | 40 36, 50 | GN -, HTN | 50 2-187 | - - | - - | 20 35, 50 |
| Cheng 1994[74]  China | CS  Zn | S  FLAAS | 13  55, 38 | -  - | 46  41 SD | -  - | Non-renal patients  - | 12  59, 42 |
| Hasanoglu 1994[75] Turkey | CS  Cu, Zn | Pl ETAAS | 27 >35, 70 | - - | - - | - - | - - | 9 >35, 67 |
| Loughrey 1994[76] United Kingdom | CS  Se | S AAS | 15 57, 47 | - IHD | 15 >2 | Cellulose or PAN - | - - | 15 44, 53 |
| Antos 1993[77] Croatia | CS  Se | S - | 45 range 32-77, 64 | - - | - - | - - | - - | 202 range 20-80, 52 |
| Colleoni 1993[78] Italy | CS  Pb | WB ETAAS | 115 51, 64 | GN, PCKD, Other - | 106 - | Cellulose - | Reference - | 383 - |
| De Kimpe 1993[79] Belgium | CS  As | S RNAA | 7 63, 43 | GN, PCKD, Other - | 57 28-126 | H/PS - | Reference  - | 6 - |
| Girelli 1993[80] Italy | CS  Se | S HGAAS | 15 58, 33 | GN, PCKD, Other None | 64 13-165 | - - | Blood donors - | 45 57, 78 |
| Holtkamp 1993[81] Germany | PC  Zn | S AAS | 65 63, - | - - | 28  >6 | C - | Non-renal patients - | 76 - |
| Hosokawa 1993[82] Japan | CS  Mn, Ni, Zn | S FAAS | 110 51, 28 | - - | 6 - | - - | - - | 50 50, 25 |
| Mayer 1993[83] Austria | CS  As | S AAS | 84 -, 51 | DM, HTN, Other  - | - 1-18 y | - - | Volunteers - | 25 -, 48 |
| Shu 1993[84] Taiwan | CS  Zn | S - | 20 34, 45 | - - | 39 - | - - | Staff - | 15 36, 73 |
| Mihailovic 1992[85] Yugoslavia | CS  Se | Pl FM | 15 59, 53 | - Other | 50 2 mo-10 y | - - | Geographic volunteers - | 19 range 25-60, 74 |
| Milly 1992[86] USA | CS  Se | WB, S, Pl GFAAS | 10 Matched | - - | - - | C - | Geographic volunteers - | 10 Matched |
| Navarro 1992[87] Venezuela | CS  Pb, V | WB GFAAS | 18 33, 78 | - - | 40 2-15 | - - | Non-renal patients - | 9 - |
| Turan 1992[88] Turkey | CS  Cd, Se | S GFAAS | 30 32, 73 | - - | - - | - - | - - | 35 28, 100 |
| Kouw 1991[89] Netherlands | PC  Zn | Pl AAS | 7 59, - | GN, Other  - | 53 23-114 | - - | Volunteers - | 14 31, - |
| Richard 1991[90] France | CS  Cu, Mn, Se, Zn | Pl ETAAS | 17 range 20-61, - | - - | - - | - - | Volunteers - | 14 range 20-62, - |
| Clyne 1990[91] Sweden | CS  Co | S NA | 7 - | - - | - - | - - | Volunteers - | 9 range 26-60, 44 |
| Kostakopoulos 1990[92] Greece | CS  Se | S - | 182 43, 48 | - - | - - | - - | Blood donors - | 100 range 41-60, 50 |
| Romero 1990[93] Venezuela | CS  Cu, Pb, Zn | WB, S, Pl GFAAS | 50 36, 60 | - - | - - | - - | Volunteers - | 52 29, 50 |
| Togni 1990[94] Italy | PC  Zn | S AAS | 25 38, 60 | GN, PCKD, HTN, Other  - | 28 4-147 | - - | Staff - | 25 38, 60 |
| Tsukamoto 1990[95] Japan | CS  V | S ETAAS | 43 55, 53 | - - | 12 1-148 | - - | - - | 39 35, 64 |
| Agenet 1989[96] France | CS  Cu, Zn | Pl AAS | 90 range 20-83, 59 | - - | - 9-192 | CA - | - - | 15 - |
| Hachache 1989[97] France | PC  Zn | S ETAAS | 33 -, 73 | GN, Other - | - 8-87 | C - | - - | 89 - |
| Hopfer 1989[98] Canada | CS  Ni | S ETAAS | 72 -, 41 | - - | - - | Acetonitrile, CR - | Staff - | 65 -, 40 |
| Ishida 1989[99] Japan | CS  V | S ETAAS | 11 - | - - | - 1mo- 18 y | - - | Geographic volunteers - | 64 34, 61 |
| Mahajan 1989[100] USA | CS  Zn | Pl AAS | 10 range 42-65, - | GN, Other None | - >6 | - - | Reference - | 5 Matched, - |
| Navarro 1989[101] Venezuela | CS  Cu, Pb, Zn | WB GFAAS | 11 39, 82 | GN, Other  - | 12 1 mo- 6.4 y | C - | - - | 69 39, 82 |
| Nixon 1989[102] USA | CS  Ni | S GFAAS | 27 - | - - | - - | - - | Reference - | 38 range 24-53 , 50 |
| Saint-Georges 1989[103] France | CS  Se | Pl  ETAAS | 39 55, 59 | - - | 8.3 y 1-17 y | C, CA, PS, PAN - | Staff - | 15 - |
| Sampson 1989[104] United Kingdom | CS  Pb | WB AAS | 107 51, 61 | - HTN | 6.3 < 1-21 y | - - | Staff - | 31 35, 39 |
| Travaglini 1989[105] Italy | CS  Zn | S AAS | 9 44, 100 | GN, PCKD, Other - | 6 y 1-19 y | - - | - - | 50 44, 100 |
| Abu-Hamdan 1988[106] USA | PC  Zn | Pl AAS | 7 47, 86 | GN, HTN, DM, Other - | 21 4-75 | - - | - - | 7 - |
| Foote 1988[107] United Kingdom | PC  Zn | Pl FLAAS | 9 range 25-60, 100 | GN, PCKD, Other  - | - >1 y | - - | - - | 13 range 22-45, 46 |
| Kuroda 1988[108] Japan | CS  Se | S ETAAS | 60 49, - | - - | - - | - - | Volunteers - | 240 42, 75 |
| Mendes 1988[109] Brazil | CS  Zn | S AAS | 41 37, 67 | GN, PCKD, HTN, Other  - | - >12 | C, CA - | - - | 25 -, 60 |
| Sondheimer 1988[110] USA | CS  Cu | Pl FLAAS | 20 49, 85 | - - | - - | - - | - - | 20 46, 100 |
| Chen 1987[111] Taiwan | CS  Zn | Pl AAS | 16 50, 31 | - - | 47 12-111 | C, CR - | - - | 48 49, 48 |
| Dworkin 1987[112] USA | CS  Se | WB, Pl FM | 24 51, 50 | - - | 75 1 wk -153 mo | - - | Staff - | 29 31, 62 |
| Foote 1987 A[113] United Kingdom | CS  Zn | S ETAAS | 20 range 25-58, 100 | - - | - >1 y | - - | Volunteers - | 86 range 18-62, 100 |
| Foote 1987 B[114] United Kingdom | CS  Se | Pl HGAAS | 29 45, 100 | - - | - >1 y | - - | Reference - | 25 41, 100 |
| Hosokawa 1987[115] Japan | CS  Ni, Zn | S AAS | 30 42, 43 | - - | 50 - | C, CA - | - - | 30 - |
| Ruiz Alcantarilla 1987[116] Spain | PC  Zn | Pl AAS | 56 44, 36 | - - | 45 - | C - | Volunteers - | 20 42, 30 |
| Sanada 1987[117] Japan | PC  Zn | S AAS | 19 41, 32 | - - | 7.3 y - | - - | - - | 75 40, 51 |
| Shu 1987[118] Taiwan | CS  Zn | Pl AAS | 54 44, 57 | - - | - < 12, ≥ 12 | - - | - - | 30 30, 63 |
| Abu-Hamdan 1986[119] USA | CS  Zn | Pl AAS | 6 45, 100 | GN, PCKD, HTN, Other - | - >2 y | - - | - - | 6 range 27-45, 100 |
| Chen 1986[120] China | CS  Cu | S AAS | 16 50, 31 | - - | 47 12-111 | C, CR - | - - | 40 53, 45 |
| Hosokawa 1986[121] Japan | CS  Zn | Pl FAAS | 15 41, 47 | - - | 35 - | - - | - - | 20 - |
| Mauras 1986[122] France | CS  B | Pl ICP/ES | 32 range 22-68, 66 | - - | - - | C, PAN - | - - | 19 range 22-35, 53 |
| Drazniowsky 1985[123] United Kingdom | CS  Ni | S FAAS | 25 - | - - | - - | - - | - - | 71 range 19-64, 55 |
| Hosokawa 1985 A[124] Japan | CS  Cu, Zn | S FAAS | 15 50, 67 | - - | - - | - - | Volunteers - | 30 - |
| Hosokawa 1985 B[125] Japan | PC  Cu | S FAAS | 42 44, 57 | - - | 56 - | C, CA, PMMA - | - - | 20 - |
| Kallistratos 1985[126] Greece | CS  Se | S FM | 27 53, 81 | PCKD, Other - | 43 4-136 | - - | - - | 40 29, 60 |
| Wills 1985[127] USA | CS  Ni | S ETAAS | 28 58, 68 | - - | 36 3-110 | - - | Volunteers - | 50 - |
| Minami 1984[128] Japan | CS  Cr | S AAS | 60 range 23-62, 40 | - - | 15 - | - - | - - | 42 20-57, 43 |
| Piechota 1983[129] Poland | CS  Cu, Zn | Pl FLAAS | 13 -, 69 | - - | - >1 y | - - | Blood donors, Staff - | 20 Matched |
| Thomson 1983[130] Australia | CS  Cd, Cr, Cu, Pb, Mn, Zn | WB, Pl FLAAS | 44 34, 63 | - - | 15 3-52 | - - | - - | 162 38, 50 |
| Mahajan 1982[131] USA | RCT nested PC  Zn | Pl AAS | 24 48, - | GN, DM, Other - | 35 >6 | - - | - - | - Matched |
| Okuyama 1982[132] Japan | CS  Cu, Zn | S AAS | 13 50, 62 | Other - | 2 y and 2 mo - | - - | Staff - | 8 35, 88 |
| Temes-Mondes 1982[133] Spain | CS  Zn | S AAS | 32 35, 53 | - - | - - | C - | - - | 103 - |
| Paniagua-Sierra 1981[134] Mexico | CS  Cu, Zn | Pl AAS | 11 34, 64 | GN, PCKD, Other - | - - | C - | Volunteers - | 13 - |
| Schiffl 1980[135]  Switzerland | CS  F | S  Fluoride activity electrode | 7  - | -  - | -  - | -  - | -  - | 8  - |
| Tsukamoto 1980[136] Japan | PC  Cu, Se, Zn | Pl FA or FAAS | 24 42, 38 | - - | 21 3-68 | - - | Volunteers - | 19 32, 53 |
| Cornelis 1979[137]  Belgium | CS  Se, Zn | S  NA | 10  50, 40 | GN, PCKD, Other  - | -  - | PAN, C  high, low | -  - | 36  -, 53 |
| Mahajan 1979[138] USA | CS  Zn | Pl AAS | 10 48, - | - - | - - | CA - | - - | 20 42, - |
| Marumo 1979[139]  Japan | CS  Cu, Se, Mn, Zn | Pl FAAS, AAS | 24 42, - | - - | 21 mean 3- 68 | - - | Volunteers - | 14 39, - |
| Mountokalakis 1979[140] Greece | CS  Zn | Pl AAS | 18 range 22-70, 39 | - - | - 1-72 | - - | - - | 34 range 15-59, 44 |
| Zumkley 1979[141] Germany | CS  Cu | Pl AAS | 52, 68 - | - - | - - | - - | - - | 14, 19 - |
| Mahajan 1978[142] USA | CS  Zn | Pl AAS | 10 - | - - | - >1y | - - | Volunteers, Non-renal patients - | 50 - |
| Willden 1974[143] England | CS  Cd | WB AAS | 21 - | - - | - - | - - | - - | 18 - |
| Rudolph 1973[144] USA | CS  B, Cu | S X-ray fluorescence | 20 - | - - | - - | - - | - - | 8 - |
| Rose 1972[145] England | CS  Zn | WB, Pl AAS | 22, 71, 29 -, 32, 52, 48 | - - | - - | - - | Staff - | 20, 22, 13 -, 70, 55,54 |
| Barbour 1971[146] United States | CS  Cu | Pl ES | 32 - | - - | - 1 mo - 3 y | C - | Staff - | 9 - |
| Mahler 1971[147] USA | CS  Cu, Zn | Pl AAS | 13 -, 100 | - - | - - | - - | Staff - | 27 - |
| Mansouri 1970[148] USA | CS  Cu, Zn | Pl AAS | 15 45, 100 | GN, Other - | - 2-47 | - - | - - | 27 - |
| Zazgornik 1971[149] Austria | CS  Zn | Pl AAS | 9 range 22-44, 56 | - - | - - | Cellophane - | - - | 30 range 23-65, - |

*Mean

Samples: WB= whole blood, Pl=plasma, S=serum

Study designs: CS=cross sectional, PC=prospective cohort, RCT=randomized controlled trial

Techniques: AAS=atomic absorption spectroscopy, ES= emission spectroscopy, ETAAS=electrothermal atomic absorption spectroscopy, FLAAS=flame atomic absorption spectroscopy, FAAS=flameless atomic absorption spectroscopy, FA=fluorescence analysis, FM=fluorometric method, GFAAS=graphite furnace atomic absorption spectroscopy, HGAAS=hydride generation atomic absorption spectroscopy, ICP/ES=inductively coupled plasma emission spectroscopy, ICP/MS=inductively coupled plasma mass spectroscopy, NA=neutron activation, RNAA=radiochemical neutron activation atomic spectroscopy

Causes of ESRD: DM=diabetes mellitus, GN=glomerulonephritis/autoimmune disease, PCKD=polycystic kidney disease, HTN=hypertension

Membrane: CA=cellulose acetate, CR=cuprammonium rayon, C=cuprophan, H=hemophan, PAN=polyacrylonitrile, PA=Polyamide, PMMA=polymethylmethacrylate, PS=polysulfone, PV=polyvinyl

Co-morbidities: NDM=non-diabetes mellitus, IHD=ischemic heart disease

Appendix Table 2. Quality assessment of included studies

|  |  |  |  |  |  |  |  |  |  |  |  |
| --- | --- | --- | --- | --- | --- | --- | --- | --- | --- | --- | --- |
|  |  |  |  |  |  |  |  |  |  |  |  |
|  |  |  |  |  |  |  |  |  |  |  |  |
|  |  |  |  |  |  |  |  |  |  |  |  |
|  |  |  |  |  |  |  |  |  |  |  |  |
|  |  |  |  |  |  |  |  |  |  |  |  |
|  |  |  |  |  |  |  |  |  |  |  |  |
|  |  |  |  |  |  |  |  |  |  |  |  |
|  |  |  |  |  |  |  |  |  |  |  |  |
|  |  |  |  |  |  |  |  |  |  |  |  |
|  |  |  |  |  |  |  |  |  |  |  |  |
|  |  |  |  |  |  |  |  |  |  |  |  |
|  |  |  |  |  |  |  |  |  |  |  |  |
|  |  |  |  |  |  |  |  |  |  |  |  |
|  |  |  |  |  |  |  |  |  |  |  |  |
|  |  |  |  |  |  |  |  |  |  |  |  |
|  |  |  |  |  |  |  |  |  |  |  |  |
|  |  |  |  |  |  |  |  |  |  |  |  |
|  |  |  |  |  |  |  |  |  |  |  |  |
|  |  |  |  |  |  |  |  |  |  |  |  |
|  |  |  |  |  |  |  |  |  |  |  |  |
|  |  |  |  |  |  |  |  |  |  |  |  |
|  |  |  |  |  |  |  |  |  |  |  |  |
|  |  |  |  |  |  |  |  |  |  |  |  |
|  |  |  |  |  |  |  |  |  |  |  |  |
|  |  |  |  |  |  |  |  |  |  |  |  |
|  |  |  |  |  |  |  |  |  |  |  |  |
|  |  |  |  |  |  |  |  |  |  |  |  |
|  |  |  |  |  |  |  |  |  |  |  |  |
|  |  |  |  |  |  |  |  |  |  |  |  |
|  |  |  |  |  |  |  |  |  |  |  |  |
|  |  |  |  |  |  |  |  |  |  |  |  |
|  |  |  |  |  |  |  |  |  |  |  |  |
|  |  |  |  |  |  |  |  |  |  |  |  |
|  |  |  |  |  |  |  |  |  |  |  |  |
|  |  |  |  |  |  |  |  |  |  |  |  |
|  |  |  |  |  |  |  |  |  |  |  |  |
|  |  |  |  |  |  |  |  |  |  |  |  |
|  |  |  |  |  |  |  |  |  |  |  |  |
|  |  |  |  |  |  |  |  |  |  |  |  |
|  |  |  |  |  |  |  |  |  |  |  |  |
|  |  |  |  |  |  |  |  |  |  |  |  |
|  |  |  |  |  |  |  |  |  |  |  |  |
|  |  |  |  |  |  |  |  |  |  |  |  |
|  |  |  |  |  |  |  |  |  |  |  |  |
|  |  |  |  |  |  |  |  |  |  |  |  |
|  |  |  |  |  |  |  |  |  |  |  |  |
|  |  |  |  |  |  |  |  |  |  |  |  |
|  |  |  |  |  |  |  |  |  |  |  |  |
|  |  |  |  |  |  |  |  |  |  |  |  |
|  |  |  |  |  |  |  |  |  |  |  |  |
|  |  |  |  |  |  |  |  |  |  |  |  |
|  |  |  |  |  |  |  |  |  |  |  |  |
|  |  |  |  |  |  |  |  |  |  |  |  |
|  |  |  |  |  |  |  |  |  |  |  |  |
|  |  |  |  |  |  |  |  |  |  |  |  |
|  |  |  |  |  |  |  |  |  |  |  |  |
|  |  |  |  |  |  |  |  |  |  |  |  |
|  |  |  |  |  |  |  |  |  |  |  |  |
|  |  |  |  |  |  |  |  |  |  |  |  |
|  |  |  |  |  |  |  |  |  |  |  |  |
|  |  |  |  |  |  |  |  |  |  |  |  |
|  |  |  |  |  |  |  |  |  |  |  |  |
|  |  |  |  |  |  |  |  |  |  |  |  |
|  |  |  |  |  |  |  |  |  |  |  |  |
|  |  |  |  |  |  |  |  |  |  |  |  |
|  |  |  |  |  |  |  |  |  |  |  |  |
|  |  |  |  |  |  |  |  |  |  |  |  |
|  |  |  |  |  |  |  |  |  |  |  |  |
|  |  |  |  |  |  |  |  |  |  |  |  |
|  |  |  |  |  |  |  |  |  |  |  |  |
|  |  |  |  |  |  |  |  |  |  |  |  |
|  |  |  |  |  |  |  |  |  |  |  |  |
|  |  |  |  |  |  |  |  |  |  |  |  |
|  |  |  |  |  |  |  |  |  |  |  |  |
|  |  |  |  |  |  |  |  |  |  |  |  |
|  |  |  |  |  |  |  |  |  |  |  |  |
|  |  |  |  |  |  |  |  |  |  |  |  |
|  |  |  |  |  |  |  |  |  |  |  |  |
|  |  |  |  |  |  |  |  |  |  |  |  |
|  |  |  |  |  |  |  |  |  |  |  |  |
|  |  |  |  |  |  |  |  |  |  |  |  |
|  |  |  |  |  |  |  |  |  |  |  |  |
|  |  |  |  |  |  |  |  |  |  |  |  |
|  |  |  |  |  |  |  |  |  |  |  |  |
|  |  |  |  |  |  |  |  |  |  |  |  |
|  |  |  |  |  |  |  |  |  |  |  |  |
|  |  |  |  |  |  |  |  |  |  |  |  |
|  |  |  |  |  |  |  |  |  |  |  |  |
|  |  |  |  |  |  |  |  |  |  |  |  |
|  |  |  |  |  |  |  |  |  |  |  |  |
|  |  |  |  |  |  |  |  |  |  |  |  |
|  |  |  |  |  |  |  |  |  |  |  |  |
|  |  |  |  |  |  |  |  |  |  |  |  |
|  |  |  |  |  |  |  |  |  |  |  |  |
|  |  |  |  |  |  |  |  |  |  |  |  |
|  |  |  |  |  |  |  |  |  |  |  |  |
|  |  |  |  |  |  |  |  |  |  |  |  |
|  |  |  |  |  |  |  |  |  |  |  |  |
|  |  |  |  |  |  |  |  |  |  |  |  |
|  |  |  |  |  |  |  |  |  |  |  |  |
|  |  |  |  |  |  |  |  |  |  |  |  |
|  |  |  |  |  |  |  |  |  |  |  |  |
|  |  |  |  |  |  |  |  |  |  |  |  |
|  |  |  |  |  |  |  |  |  |  |  |  |
|  |  |  |  |  |  |  |  |  |  |  |  |
|  |  |  |  |  |  |  |  |  |  |  |  |
|  |  |  |  |  |  |  |  |  |  |  |  |
|  |  |  |  |  |  |  |  |  |  |  |  |
|  |  |  |  |  |  |  |  |  |  |  |  |
|  |  |  |  |  |  |  |  |  |  |  |  |
|  |  |  |  |  |  |  |  |  |  |  |  |
|  |  |  |  |  |  |  |  |  |  |  |  |
|  |  |  |  |  |  |  |  |  |  |  |  |
|  |  |  |  |  |  |  |  |  |  |  |  |
|  |  |  |  |  |  |  |  |  |  |  |  |
|  |  |  |  |  |  |  |  |  |  |  |  |
|  |  |  |  |  |  |  |  |  |  |  |  |
|  |  |  |  |  |  |  |  |  |  |  |  |
|  |  |  |  |  |  |  |  |  |  |  |  |
|  |  |  |  |  |  |  |  |  |  |  |  |
|  |  |  |  |  |  |  |  |  |  |  |  |
|  |  |  |  |  |  |  |  |  |  |  |  |
|  |  |  |  |  |  |  |  |  |  |  |  |
|  |  |  |  |  |  |  |  |  |  |  |  |
|  |  |  |  |  |  |  |  |  |  |  |  |
|  |  |  |  |  |  |  |  |  |  |  |  |
|  |  |  |  |  |  |  |  |  |  |  |  |
|  |  |  |  |  |  |  |  |  |  |  |  |
