# Supplementary material for: Trace elements in hemodialysis patients: a systematic review and meta-analysis
Source: BMC Med. 2009 May 19;7:25. doi: 10.1186/1741-7015-7-25 (PMC2698829; doi:10.1186/1741-7015-7-25)
Supplement: Additional file 2 — Appendix Table S1 and S2. Table S1 – Description of included studies. Table S2 – Quality assessment of included studies. [file 1741-7015-7-25-S2.doc]

Appendix Table 2. Quality assessment of included studies

| Study | Study objectives | Selection criteria | Time on dialysis | Technique(s) | Measurement error | Adjustment for age and sex | When controls accrued | Where controls accrues | Sample size calculation | Distribution of age and sex | Confidence intervals or ranges |
| --- | --- | --- | --- | --- | --- | --- | --- | --- | --- | --- | --- |
| Bober 2007[24] | Y | I | N | Y | N | N | N | N | N | N | Y |
| Zagrodzki 2007[25] | Y | I | Y | Y | Y | N | N | N | N | P | Y |
| Batista 2006[26] | Y | A | Y | Y | Y | N | N | Y | N | Y | Y |
| Fellah 2006[27] | Y | A | P | Y | N | Y | N | N | N | Y | Y |
| Hsieh 2006[28] | Y | P | P | Y | N | P | N | Y | N | P | Y |
| Kim 2006[29] | Y | A | Y | Y | N | Y | N | Y | N | Y | Y |
| Menevse 2006[31] | Y | A | P | Y | N | N | N | N | N | P | Y |
| Navarro-Alarcon 2006[30] | Y | P | N | Y | Y | N | N | Y | N | P | Y |
| Yilmaz 2006[32] | Y | A | P | Y | N | P | N | N | N | Y | Y |
| Alabdullah 2005[33] | Y | P | N | Y | N | N | N | Y | N | N | Y |
| Bober 1995[34] | N | I | N | Y | N | N | N | N | N | P | Y |
| Bozalioglu 2005[35] | Y | P | P | Y | N | P | N | N | N | P | Y |
| Cabral 2005[36] | Y | A | N | Y | N | N | N | N | N | Y | Y |
| Sandhu 2005[37] | Y | A | N | Y | N | Y | N | N | N | Y | Y |
| Ribeiro 2004[38] | Y | A | Y | Y | N | N | N | Y | N | P | Y |
| Yavuz 2004[39] | Y | A | Y | Y | N | P | Y | Y | N | Y | Y |
| Zachara 2004[40] | Y | P | N | Y | Y | N | N | N | N | P | Y |
| Adamowicz 2002[41] | Y | I | N | Y | N | N | N | N | N | P | Y |
| Candan 2002[42] | Y | A | N | Y | N | Y | N | N | N | P | Y |
| Pietrzak 2002[43] | Y | I | Y | Y | N | N | N | N | N | P | Y |
| Torra 2002[44] | Y | P | N | Y | Y | Y | N | N | N | N | Y |
| Muniz 2001[45] | Y | I | N | Y | N | N | N | Y | N | N | Y |
| Weissgarten 2001[46] | Y | P | P | Y | N | Y | N | N | N | N | Y |
| Zachara 2001[47] | Y | P | Y | Y | Y | N | N | N | N | P | Y |
| Bogye 2000[48] | Y | A | N | Y | N | P | N | Y | N | N | Y |
| Krizek 2000[49] | Y | I | Y | Y | Y | N | N | N | N | P | Y |
| Lee 2000[50] | Y | A | P | Y | N | Y | N | Y | N | P | Y |
| Mestek 2000[51] | Y | I | N | Y | Y | N | N | Y | N | N | Y |
| Roxborough 2000 [52] | Y | I | N | Y | Y | P | N | N | N | P | Y |
| Hwang 1999[53] | Y | I | Y | Y | Y | N | N | Y | N | P | Y |
| Bonforte 1998[54] | Y | I | N | Y | N | N | N | N | N | N | Y |
| Chataut 1998[55] | Y | A | N | Y | N | N | N | N | N | P | Y |
| Nordio 1998[56] | Y | I | N | Y | N | N | N | N | N | P | Y |
| Turk 1998[57] | Y | I | Y | Y | N | N | N | N | N | P | Y |
| Zhang 1998[58] | Y | P | N | Y | Y | N | Y | Y | N | N | Y |
| Zima 1998 A[2] | Y | I | N | Y | N | N | N | Y | N | P | Y |
| Zima 1998 B[59] | Y | I | N | Y | N | Y | N | N | N | N | Y |
| Hung 1997[60] | Y | A | P | Y | N | P | N | Y | N | Y | Y |
| Iotova 1997[61] | Y | I | Y | Y | N | N | N | N | N | P | Y |
| Koenig 1997[62] | Y | I | Y | Y | N | N | N | N | N | N | Y |
| Bonomini 1996[63] | Y | A | Y | Y | Y | Y | N | Y | N | P | Y |
| Emenaker 1996[64] | Y | P | N | Y | N | Y | N | N | N | P | Y |
| Gunduz 1996[65] | Y | P | Y | Y | N | N | N | N | N | P | Y |
| Lin 1996[66] | Y | A | Y | Y | Y | N | N | N | N | P | Y |
| Marchante-Gayon 1996[67] | Y | I | N | Y | Y | N | N | N | N | N | Y |
| Rashid 1996[68] | N | I | P | Y | Y | N | N | Y | N | P | Y |
| Romero 1996[69] | Y | I | Y | Y | Y | N | N | N | N | Y | Y |
| Usuda 1996[16] | Y | P | P | Y | Y | Y | N | Y | N | P | Y |
| Yoshimura 1996[70] | Y | P | P | Y | N | P | N | Y | N | P | Y |
| Bonomini 1995[71] | Y | A | Y | Y | Y | Y | N | N | N | P | Y |
| Granadillo 1995 A[72] | Y | P | Y | Y | Y | N | N | N | N | P | Y |
| Granadillo 1995 B[73] | Y | A | Y | Y | Y | Y | Y | N | N | P | Y |
| Cheng 1994[74] | Y | P | N | Y | N | N | N | N | N | Y | Y |
| Hasanoglu 1994[75] | Y | I | N | Y | N | N | N | N | N | N | Y |
| Loughrey 1994[76] | Y | A | Y | Y | N | N | N | N | N | N | Y |
| Antos 1993[77] | Y | P | N | Y | N | Y | N | N | N | P | Y |
| Colleoni 1993[78] | Y | P | Y | Y | Y | N | N | N | N | N | Y |
| De Kimpe 1993[79] | Y | I | Y | Y | N | N | N | N | N | N | Y |
| Girelli 1993[80] | Y | A | Y | Y | Y | P | Y | Y | N | P | Y |
| Holtkamp 1993[81] | N | I | Y | Y | N | N | N | Y | N | N | Y |
| Hosokawa 1993[82] | Y | A | Y | Y | N | N | N | N | N | N | Y |
| Mayer 1993[83] | N | I | P | Y | Y | P | N | N | N | P | Y |
| Shu 1993[84] | Y | A | Y | Y | N | N | N | N | N | P | Y |
| Mihailovic 1992[85] | Y | P | Y | Y | N | N | N | Y | N | P | Y |
| Milly 1992[86] | Y | I | N | Y | Y | Y | N | Y | N | N | Y |
| Navarro 1992[87] | Y | P | Y | Y | Y | N | N | N | N | N | Y |
| Turan 1992[88] | Y | Y | N | Y | N | N | N | N | N | P | Y |
| Kouw 1991[89] | Y | P | Y | Y | Y | N | N | N | N | P | Y |
| Richard 1991[90] | Y | P | P | Y | Y | N | N | N | N | N | Y |
| Clyne 1990[91] | Y | I | N | Y | N | N | N | N | N | P | Y |
| Kostakopoulos 1990[92] | Y | I | N | N | N | N | N | Y | N | P | Y |
| Romero 1990[93] | Y | I | N | Y | N | N | N | N | N | P | Y |
| Togni 1990[94] | Y | I | Y | Y | Y | Y | N | Y | N | P | Y |
| Tsukamoto 1990[95] | N | P | Y | Y | N | N | N | Y | N | P | Y |
| Agenet 1989[96] | Y | I | P | Y | N | N | N | N | N | N | Y |
| Hachache 1989[97] | Y | P | P | Y | N | N | N | N | N | N | Y |
| Hopfer 1989[98] | Y | A | N | Y | N | N | N | Y | N | N | Y |
| Ishida 1989[99] | Y | I | P | Y | Y | N | N | N | N | P | Y |
| Mahajan 1989[100] | Y | P | P | Y | N | P | N | N | N | P | Y |
| Navarro 1989[101] | Y | I | Y | Y | N | Y | N | N | N | N | Y |
| Nixon 1989[102] | Y | I | N | Y | Y | N | N | N | N | N | Y |
| Saint-Georges 1989[103] | Y | I | Y | Y | N | N | N | N | N | N | Y |
| Sampson 1989[104] | Y | A | Y | Y | Y | Y | N | Y | N | P | Y |
| Travaglini 1989[105] | Y | I | Y | Y | Y | Y | N | N | N | Y | Y |
| Abu-Hamdan 1988[106] | Y | P | Y | Y | N | N | N | N | N | P | Y |
| Foote 1988[107] | Y | P | P | Y | Y | N | N | N | N | N | Y |
| Kuroda 1988[108] | Y | P | N | Y | Y | Y | N | Y | N | Y | Y |
| Mendes 1988[109] | Y | I | N | Y | N | N | N | N | N | N | Y |
| Sondheimer 1988[110] | Y | A | P | Y | N | Y | N | N | N | P | Y |
| Chen 1987[111] | Y | I | Y | Y | Y | N | N | N | N | P | Y |
| Dworkin 1987[112] | Y | I | N | Y | Y | Y | N | Y | N | P | Y |
| Foote 1987 A[113] | Y | P | P | Y | N | P | N | N | N | N | Y |
| Foote 1987 B[114] | Y | P | P | Y | Y | N | N | N | N | N | Y |
| Hosokawa 1987[115] | Y | P | Y | Y | N | N | N | N | N | N | Y |
| Ruiz Alcantarilla 1987[116] | Y | I | Y | Y | N | N | N | N | N | P | Y |
| Sanada 1987[117] | Y | P | P | Y | N | N | N | N | N | P | Y |
| Shu 1987[118] | Y | I | N | Y | Y | N | Y | N | N | P | Y |
| Abu-Hamdan 1986[119] | Y | A | P | Y | N | N | N | N | N | P | Y |
| Chen 1986[120] | Y | P | Y | Y | Y | N | N | N | N | P | Y |
| Hosokawa 1986[121] | Y | P | Y | Y | Y | N | N | N | N | N | Y |
| Mauras 1986[122] | N | P | N | Y | Y | P | N | N | N | P | Y |
| Drazniowsky 1985[123] | Y | I | N | Y | N | N | N | N | N | N | Y |
| Hosokawa 1985 A[124] | Y | P | N | Y | N | N | N | N | N | N | Y |
| Hosokawa 1985 B[125] | Y | P | Y | Y | Y | N | N | N | N | N | Y |
| Kallistratos 1985[126] | N | P | Y | Y | Y | N | N | N | N | P | Y |
| Wills 1985[127] | Y | P | Y | Y | N | N | N | N | N | P | Y |
| Minami 1984[128] | Y | P | P | Y | N | N | N | N | N | P | Y |
| Piechota 1983[129] | Y | I | P | Y | N | Y | N | Y | N | Y | Y |
| Thomson 1983[130] | Y | I | Y | Y | N | N | Y | N | N | P | Y |
| Mahajan 1982[131] | N | P | Y | Y | N | Y | N | N | N | Y | Y |
| Okuyama 1982[132] | Y | P | Y | Y | N | N | N | Y | N | P | Y |
| Temes-Montes 1982[133] | Y | P | N | Y | N | N | N | N | N | N | N |
| Paniagua-Sierra 1981[134] | Y | I | N | Y | N | N | N | N | N | N | N |
| Schiffl 1980[135] | Y | I | N | Y | Y | N | N | N | N | N | Y |
| Tsukamoto 1980[136] | Y | I | Y | Y | N | N | N | N | N | P | Y |
| Cornelis 1979[137] | Y | I | N | Y | N | N | N | N | N | N | Y |
| Mahajan 1979[138] | Y | I | P | Y | N | Y | N | N | N | P | Y |
| Marumo 1979[139] | Y | I | Y | Y | N | N | N | N | N | P | Y |
| Mountokalakis 1979[140] | Y | I | P | Y | N | N | N | N | N | P | Y |
| Zumkley 1979[141] | Y | I | N | Y | N | N | N | N | N | N | Y |
| Mahajan 1978[142] | Y | I | P | Y | N | Y | N | Y | N | N | Y |
| Willden 1974[143] | N | I | N | Y | N | N | N | N | N | N | Y |
| Rudolph 1973[144] | Y | A | N | Y | N | N | N | Y | N | N | Y |
| Rose 1972[145] | Y | P | N | Y | N | N | N | Y | N | N | Y |
| Barbour 1971[146] | Y | P | P | Y | N | N | N | Y | N | N | Y |
| Mahler 1971[147] | Y | I | N | Y | N | N | N | Y | N | N | Y |
| Mansouri 1970[148] | Y | I | Y | Y | N | N | N | N | N | N | Y |
| Zazgornik 1970[149] | Y | P | N | Y | N | N | N | N | N | P | Y |

A=adequate, I=inadequate, P=partial, N=no, Y=yes
